# The Angiosperm Stem Hemiparasitic Genus *Cassytha* (Lauraceae) and Its Host Interactions: A Review

**DOI:** 10.3389/fpls.2022.864110

**Published:** 2022-06-06

**Authors:** Hongxiang Zhang, Singarayer Florentine, Kushan U. Tennakoon

**Affiliations:** ^1^Institute of Innovation, Science and Sustainability, Future Regions Research Centre, Federation University, Berwick, VIC, Australia; ^2^Institute of Geography and Agroecology, Chinese Academy of Sciences, Changchun, China; ^3^Institute of Innovation, Science and Sustainability, Future Regions Research Centre, Federation University, Ballarat, VIC, Australia

**Keywords:** aerial parasite, *Cassytha filiformis*, *Cassytha pubescens*, environmental change, haustorium, plant infection, nutrient transfer

## Abstract

*Cassytha*, also known as laurel dodder or love vine, is a stem hemiparasite of the Lauraceae family. It has long been used for medicinal purposes in many countries and has increasingly influenced agricultural and natural ecosystems by its effects on a wide range of host species. Previous studies have focused on the taxonomy and evolutionary position of different *Cassytha*, with the pan-tropical species *Cassytha filiformis* being the most widely studied. However, *Cassytha*–host interactions have never been reviewed, which is an essential issue related to the understanding of mechanisms underlying plant hemiparasitic and the assessment of benefits and damage caused by aerial parasitic plants. This review explores the parasitic habits, worldwide distribution, and host range of *Cassytha*, and examines its impacts on the biology of host plants and the overall influence of environmental changes on *Cassytha*–host associations. We also comment on areas of future research directions that require to better understanding *Cassytha*–host interactions. It appeared that some traits, such as flowering phenology, facilitated *Cassytha*’s widespread distribution and successful parasitism and that *Cassytha* preferred woody species rather than herbaceous species as a host, and preferred species from certain families as hosts, such as Fabaceae and Myrtaceae. Cassytha often decreased biomass and impacted the physiology of host species and global environmental changes seemed to intensify the negative impacts of *Cassytha* on their hosts. *Cassytha* was not only a noxious weed, but can also function as a biocontrol agent to mitigate alien plant invasion.

## Introduction

Parasitism is a widespread phenomenon and an important ecological interaction, with many organisms being engaged as either parasites or hosts (Combes, 2001; Krasylenko et al., 2021). Plant parasitism includes directly parasitizing host plants and absorbing water and nutrition *via* haustorium and indirectly parasitizing other plants and acquiring nutrition *via* mycorrhizal fungi (Nickrent and Musselman, 2004; Nickrent, 2014; Krasylenko et al., 2021). These parasitic flowering plants include about 4,500 species and have been divided into 12 independent evolutionary lineages (Nickrent, 2020; Těšitel et al., 2021). Parasitic flowering plants either attach to host roots or shoots. They can be chlorophyllous and thus, capable of photosynthesis (hemiparasites) or not (holoparasites) and they can be further divided into four types: root vs. stem hemiparasites, and root *vs.* stem holoparasites (Musselman and Press, 1995; Teixeira-Costa and Davis, 2021). They can also be classified as obligate parasites (which indicates that they need a host plant to acquire nutrients to survive after germination) and facultative parasites (which are capable of reaching maturity without attachment to the host) (Shen et al., 2006). According to studies of functional diversity, parasitic flowering plants have been recognized as euphytoid parasites (resembling true plants that are capable of photosynthesis while infesting the underground root system of their hosts), mistletoes (shrubby plants with seeds that germinate autonomously and directly upon the branches of their hosts), parasitic vines (i.e., *Cassytha* and *Cuscuta*), obligate root parasites (parasites that germinate underground, often in response to host-derived chemicals, and infest the root systems of their hosts) and endoparasites (vegetative body of parasitic plants is reduced to mycelial-like strands of cells embedded within their respective host roots or stems) (Teixeira-Costa and Davis, 2021).

Parasitic flowering plants have had renewed attention over the past three decades (Nickrent, 2020), since they caused serious problems across a wide range of major ecosystems, from subarctic tundra, heathlands, savanna woodlands, deserts, temperate and tropical forests, and agricultural ecosystem (Press and Phoenix, 2005; Shen et al., 2006). Concurrently, the parasitic plants have medicinal and cultural values (Těšitel et al., 2021) and play important roles as keystone species (species that exert a disproportionate impact on community biodiversity relative to its presence in a community; Paine, 1969; Miller et al., 2003; Caraballo-Ortiz, 2019; Těšitel et al., 2021), and ecosystem engineers (species that directly and indirectly shift the availability of resources to other species in the community) in these habitats (Jones et al., 1994; Press and Phoenix, 2005; Bardgett et al., 2006; Spasojevic and Suding, 2011). Despite a large body of research on the biology of root hemiparasites regarding the Scrophulariaceae and Santalaceae species, plus, mistletoes of families Loranthaceae and Viscaceae, and the stem holoparasites *Cuscuta* (Tennakoon et al., 1997; Lanini and Kogan, 2005; Phoenix and Press, 2005; Carnegie et al., 2009; Glatzel and Geils, 2009; Mishra, 2009; Furuhashi et al., 2011; Clarke et al., 2019), a notable exception is stem hemiparasitic genus *Cassytha.* Being stem-parasitic vines, *Cassytha* and *Cuscuta* behave similarly and are often referred to together or represented inadvertently as *Cuscuta* (Weber, 1981; Mishra, 2009). However, *Cassytha* is a hemiparasite whilst *Cuscuta* is a holoparasite, and they actually differ in many aspects such as the action of the haustorium, their stem appearance, and life span (Tennakoon et al., 2016; Těšitel, 2016; Teixeira-Costa and Davis, 2021). Study on *Cassytha* has been relatively neglected, leading to it being less well characterized compared to its companion *Cuscuta* (Lanini and Kogan, 2005; Mishra, 2009).

*Cassytha*, belongs to the sub-family Cassythoideae, the family Lauraceae and the magnoliid clade (Awang et al., 2018). *Cassytha filiformis* has been exploited for medicines, cosmetics, rope-making, and cushioning in the Pacific Islands (Whistler, 1992), and is treated as an important medicinal plant both in China (Huang et al., 2021) and Nigeria (Ambi et al., 2017). *C. filiformis* and *Cassytha glabella* have been treated as sources of bush tucker and medicines by the Australian Aboriginals (Levitt, 1981), and *Cassytha pubescens* has the potential to be used as a biocontrol agent for alien invasive species in southern Australia (Těšitel et al., 2020). *Cassytha pondoensis* is recognized as a medicinal plant in Angola (Novotna et al., 2020) whilst *C. pubescens, Cassytha melantha, Cassytha racemosa, Cassytha pomiformis*, and *C. filiformis* contain alkaloids (Collins et al., 1990) and *C. filiformis, C. pubescens*, and *Cassytha capillaris* contain essential oils (Brophy et al., 2009). The *Cassytha* grouping contains 19 species (Table 1) according to The Plant List^1^, 16 of which occur in Australia. There are 13 species endemic to Australia, one being pantropical (*C. filiformis*), one extending into Assam, Borneo, Lesser Sunda Islands, Malulu, New guinea, and Vietnam (*C. capillaris*), and one also being found in New Zealand (*C. pubescens*). The other three species are endemic to Africa (*Cassytha ciliolata* and *C. pondoensis*) or Thailand (*Cassytha larsenii*) (see Weber, 1981; Weber, 2007; Kokubugata et al., 2012; Nickrent, 2020). It has been reported that *C. capillaris* also occurs in Indonesia and China (Song et al., 2017; Liu et al., 2021), which has not been confirmed. Kokubugata et al. (2012) claimed that *Cassytha pergracilis* was an endemic species found in Japan (Kokubugata and Yokota, 2012). However, it is not recorded in Global Biodiversity Information Facility^2^ and is recognized as a synonym of *C. glabella* in The Plant List. *Cassytha muelleri, Cassytha paniculata*, and *Cassytha phaeolasia* were recorded as species in Weber (2007) and in the Flora of Australia^3^ that follows the Australian Plant Census^4^, but they are treated as synonyms of *C. racemosa* and *C. pubescens*, respectively (Supplementary Appendix Table S1).

**TABLE 1:** *Cassytha s*pecies and their worldwide distributions.

	*Cassytha* species	Distribution	Habitat	Uses
1	*Cassytha aurea* J.Z.Weber	Western Australia	Coastal, woodlands	
2	*Cassytha candida* (J.Z.Weber) J.Z.Weber	Northern Territory, Western Australia	Woodlands	
3	*Cassytha capillaris* Meisn.	Assam, Borneo, Lesser Sunda Islands, Maluku, New Guinea Northern Territory, Queensland, Vietnam, Western Australia	Around the coast, tropical and subtropical moist broadleaf forests	
4	*Cassytha ciliolata* Nees	Cape Provinces	Tropical and subtropical moist broadleaf forests	
5	*Cassytha filiformis* L.	Aldabra, Andaman Islands, Angola, Bahamas, Bangladesh, Belize, Benin, Bolivia, Botswana, Brazil North, Brazil Northeast, Brazil South, Brazil Southeast, Brazil West-Central, Brunei, Burkina, Burundi, Cambodia, Cameroon, Cape Provinces, Caroline Islands, Cayman Islands, Central African Republic, Chad, Chagos Archipelago, China South-Central, China Southeast, Cocos (Keeling) Islands, Colombia, Comoros, Congo, Cook Islands, Costa Rica, Cuba, Dominican Republic, Ethiopia, Fiji, Florida, French Guiana, Gabon, Gambia, Ghana, Gilbert Islands, Guatemala, Guinea, Guinea-Bissau, Guyana, Hainan, Haiti, Hawaii, Honduras, India, Ivory Coast, Jamaica, Japan, Jawa, Kazan-retto, Kenya, KwaZulu-Natal, Laccadive Islands, Laos, Leeward Islands, Lesser Sunda Islands, Liberia, Line Islands, Madagascar, Malawi, Malaya, Maldives, Mali, Maluku, Marianas, Marquesas, Marshall Islands, Mauritius, Mozambique, Mozambique Channel I, Myanmar, Namibia, Nansei-shoto, Nauru, Netherlands Antilles, New Caledonia, New Guinea, New South Wales, Nicaragua, Nicobar Islands, Nigeria, Niue, Northern Provinces, Northern Territory, Ogasawara-shoto, Panamá, Philippines, Phoenix Islands, Pitcairn Islands, Puerto Rico, Queensland, Rodrigues, Rwanda, Réunion, Samoa, Saudi Arabia, Senegal, Seychelles, Sierra Leone, Society Islands, Solomon Islands, Somalia, South China Sea, Sri Lanka, Sulawesi, Suriname, Swaziland, Taiwan, Tanzania, Togo, Tokelau-Manihiki, Tonga, Trinidad-Tobago, Tuamotu, Tubuai Islands, Turks-Caicos Islands, Tuvalu, Uganda, Vanuatu, Venezuela, Vietnam, Wallis-Futuna Islands, Western Australia, Windward Islands, Yemen, Zambia, Zaïre, Zimbabwe	Deserts and xeric shrublands, flooded grasslands and savannas, mangroves, Mediterranean forests, woodlands and scrub, montane grasslands and shrublands, temperate conifer forests, tropical and subtropical coniferous forests, tropical and subtropical dry broadleaf forests, tropical and subtropical grasslands, savannas and shrublands, tropical and subtropical moist broadleaf forests	Cosmetics, cushioning, medicine, poison, rope-making, sources of bush tucker
6	*Cassytha flava* Nees	Western Australia		
7	*Cassytha flindersii* (J.Z.Weber) J.Z.Weber	South Australia	Mountain range, forests	
8	*Cassytha glabella* R.Br.	New South Wales, Queensland, South Australia, Tasmania, Victoria, Western Australia	Near the coast, forest, shrubland	Medicine, sources of bush tucker
9	*Cassytha larsenii* Kosterm.	Thailand	Tropical and subtropical moist broadleaf forests	
10	*Cassytha melantha* R.Br.	New South Wales, South Australia, Tasmania, Victoria, Western Australia	Around the coast and far inland	Medicinal or poisonous (containing alkaloids and essentia oils)
11	*Cassytha micrantha* Meisn.	Western Australia	Near the coast and inland to the mountain range	
12	*Cassytha nodiflora* Meisn.	Western Australia	Along the coast, sandy flats	
13	*Cassytha pedicellosa* J.Z.Weber	Tasmania	Near the coast, heathland	
14	*Cassytha peninsularis* J.Z.Weber	South Australia	Around the coast, mountain range	
15	*Cassytha pomiformis* Nees	Western Australia	Along the coast and also inland	Medicinal or poisonous (containing alkaloids and essential oils)
16	*Cassytha pondoensis* Engl.	Angola, Cape Provinces, KwaZulu-Natal, Malawi, Mozambique, Tanzania, Zambia, Zimbabwe	Around the coast, tropical and subtropical grasslands, savannas and shrublands, tropical and subtropical moist broadleaf forests	Medicine
17	*Cassytha pubescens* R.Br.	New South Wales, New Zealand North, Queensland, South Australia, Tasmania, Victoria	Along the coast, temperate broadleaf and mixed forests	Biocontrol agent, medicinal or poisonous (containing alkaloids and essential oils)
18	*Cassytha racemosa* Nees	New South Wales, Queensland, Western Australia	Along the coast	Medicinal or poisonous (containing alkaloids and essential oils)
19	*Cassytha rufa* J.Z.Weber	Queensland	Woodlands, forests	

*We used the latest version of The Plant List for species nomenclature. The distribution information was based on the Plants of the World Online (POWO) and the habitat of species was from Weber (1981, 2007), Olson and Dinerstein (2002), and POWO. The uses of species was summarized from the text of the third paragraph in Introduction.*

As a widespread pan-tropical species, *C. filiformis* has been more extensively studied than other species of this genus. However, a group of scientists from South Australia has recently investigated the potential of using native *C. pubescens* to control the alien invasive shrubs *Ulex europaeus* and *Cytisus scoparius* (Cirocco et al., 2016a, 2017, 2018; Facelli et al., 2020). For other species in the *Cassytha* genus, there are relatively a few taxonomic studies and field investigations concentrating on species in certain habitats, with few empirical studies. For example, the cuticular character of all the *Cassytha* species and the stem and systematic anatomy of *C. ciliolata, C. filiformis*, C. *glabella, C. melantha*, and *C. pubescens* has been studied (Sastri, 1962; Beaman, 1971; Awang et al., 2018). The chlorophyll content and photosynthetic characteristics of *C. ciliolata* and *C. filiformis* in South Africa (De La Harpe et al., 1979, 1980, 1981) and the seasonal fluctuations in pigment chemistry of *C. glabella* and *C. pubescens* in Australia (Close et al., 2006) have also been investigated. However, the *Cassytha*–host interactions of any of these species have not been reviewed.

We suggest that a detailed interpretation of *Cassytha*–host interactions are important to allow an understanding of their complex interactive biology and to allow us to build up a relatively detailed picture of the ecophysiological behavior of these parasite–host associations. Further, parasites have a great impact on plant communities even though they might contribute a minor component in the mix, and a single parasite may seriously influence a large portion of an ecosystem (Press and Phoenix, 2005). Hence, the understanding of *Cassytha*–host interactions can also help to control the damage induced by these parasites in both agriculture and natural settings. In addition, it may be possible to utilize these stem parasites to control invasive weeds and use them separately for raw material and medicinal purposes (Těšitel et al., 2021). In this review, we summarize currently available information on *Cassytha*–host interactions focusing on its parasitic nature and worldwide distribution, identifying host range and preference, noting the impacts of *Cassytha* on host species and understanding the overall responses to the changes in climate, viable control strategies under heavy infestations and its sustainable utilization. We also identify gaps in current knowledge of this area and suggest future study directions deemed necessary for *Cassytha*–host interactions.

## Parasitic Habits and Host Range of *Cassytha*

In this section, we mainly test whether particular habits facilitate the wide parasitism of *Cassytha* and if host species of certain life forms and/or hosts belonging to selected families are preferred.

### Life History Habits

**FIGURE 1:**
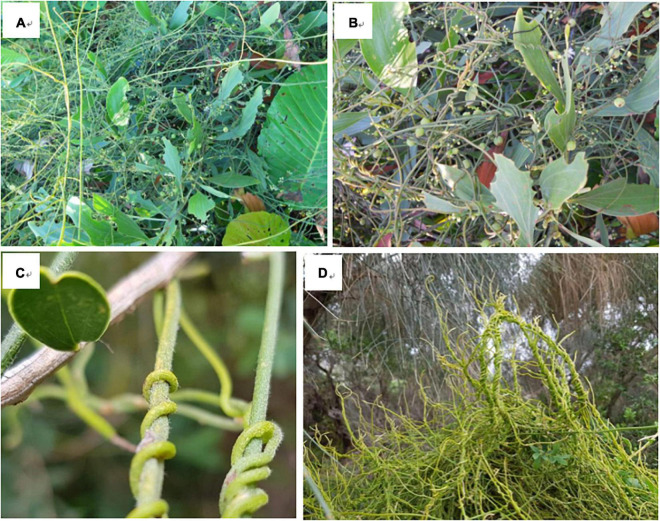
Images of *Cassytha filiformis* (**A,B**, in Brunei) and *Cassytha pubescens* (**C,D**, in Australia) on host species (source: KT).

*Cassytha* (Figure 1) has twining stems with scaly leaves (Kuijt, 1969). Half of the Australian distributed species have evidence of flowering throughout the entire year (*Cassytha aurea, Cassytha candida, C. capillaris, C. filiformis, Cassytha flava, C. glabella, C. racemosa*, and *Cassytha rufa*), whilst the other half of the species are seasonally flowering. For example, *C. melantha* flowers from June to October (Weber, 1981, 2007). The fruit developments appear to be around 2 months, such as with *Cassytha flindersii*, but the information is scarce for other species (Weber, 2007). The dispersal of *Cassytha* is mainly dependent on seeds (Tennakoon et al., 2016), and the fruit is a drupe with a single seed and a white translucent, fleshy pericarp (Mishra, 2009). Thus, it is assumed that zoochory (e.g., dispersal by vertebrates) is important for the spread of *Cassytha* (French and Westoby, 1996). Recent experiments also provide evidence that mammals are involved in the dispersal of *Cassytha pubescens* (Maciunas et al., 2022). Some *Cassytha* species, such as *C. filiformis*, have refractory seeds with a hard seed coat and are found predominantly in coastal regions (Heide-Jørgensen, 2008; Mahadevan and Jayasuriya, 2013). It has been found that the fruits of *C. filiformis* floated for months in the Pacific (Muir, 1933). These lead to an additional water-mediated dispersal hypothesis for this species (Teixeira-Costa and Davis, 2021), but further evidence is lacking.

Seeds of *C. pubescens* also have physical dormancy, which can be broken by heat and scarification of the husk, and the germination rate of heated seeds was found to be much higher than that of scarified seeds (Tsang, 2010). This suggests *C. pubescens* may have evolved fire-related germination cues with its native hosts (Tsang, 2010). *Cassytha* seeds germinate on the ground, with a rudimentary and short-lived root (McLuckie, 1924; Kuijt, 1969) and without needing any host influence. This independent (autotrophic) growth period is reported to be between one to a few weeks (McLuckie, 1924). *C. filiformis* seedlings can survive for more than 1 month prior to parasitizing a viable host, as long as there is photosynthesis, plus water and nutrient absorption from the soil by rudimentary roots. It can grow up to 30 cm in length without attaching to a host (Nelson, 2008; Furuhashi et al., 2016), even though *Cassytha* belongs to the obligate parasite class (Shen et al., 2010; Balasubramanian et al., 2014).

The flowering phenology, seed dispersal, and autotrophic habits before attaching to a host facilitate wide distribution and parasitism of *Cassytha* species. However, very little has been reported regarding the early life history of *Cassytha*, leaving us with a paucity of information related to the autotrophic stage of germinated *Cassytha* prior to establishment of successful attachments with hosts, and the cues involved with the stimulation of haustorial initiation from the *Cassytha* seedlings. It is thought, nevertheless, that all these growth and reproduction habits are closely related to host selection and parasite–host interactions.

### Haustorial Development and Attachment Mechanisms

*Cassytha* can actively move their young stems to find suitable hosts (Tennakoon et al., 2016). It is not clear whether chemical cues released by hosts trigger *Cassytha*’s attachment to hosts, in a similar manner to *Cuscuta* foraging volatile substances from host plants (Furuhashi et al., 2016; Tennakoon et al., 2016). The attachment structure of *Cassytha* on hosts is by means of the haustorium, and a single *Cassytha* has been observed to produce hundreds of haustoria (Kuijt, 1969; Groom and Lamont, 2015). Haustoria are generally produced on young shoots or leaf rachises of the host plants (Werth et al., 1979). Twining is the critical first step of attachment, but less attention has been paid compared to haustorial development. Incident light and plant hormones have been shown to control tendril coiling in laboratory conditions (Furuhashi et al., 2021). Blue light and a lower far-red/red light (FR/R) ratio were noted to be essential for twining and subsequent haustorial induction of *C. filiformis*, respectively. Regarding plant hormones, seedlings of *C. filiformis* solely with auxin or cytokinin under blue light showed twining and haustorial induction. Seedlings with the hormones brassinolide and cytokinin showed twining even under dark conditions, but brassinolide acting alone did not stimulate twining (Furuhashi et al., 2021). This observation indicates that cytokinin and auxins may be the key hormones responsible in *Cassytha* to allow twining around hosts that facilitate subsequent haustorial initiation and successful *Cassytha*–host associations.

The swelling or cushion-like haustorium of *Cassytha* has two parts; the upper haustorium that lies external to the host and the endophyte that penetrates host tissues (Heide-Jørgensen, 1991). The vertical sections of the haustorium of *C. filiformis* on the leaves of host *Canthium rheedii*, included a vascular core, interrupted zone, collapsed layer, and clasping folds. Researchers have observed graniferous tracheary cells containing granules in the vascular core of the haustorium (Rajanna and Shivamurthy, 2001). When a stem of *C. filiformis* comes into contact with the compatible host stem, the cortical cells of the hemiparasite stem divide quickly and form the upper haustorium. The cells continually elongate and protuberate inside the host, modifying into finger-like digitate cells. The digitate cells break host stem cells with mechanical pressure and then differentiate into hypha-like lower endophyte structures (Balasubramanian et al., 2014). For example, in an association between *C. filiformis* and the host plant *Morinda tinctoria*, the link initially was slack and easy to be separated; however, subsequent formation of the endophyte and its ability to penetrate the host tissue facilitated a firm haustorial connection (Balasubramanian et al., 2014). Li and Yao (1992) studied the anatomical aspects of haustorial development of *C. filiformis* attached to a *Salix purpurea* stem. They divided the process into four stages: (i) polarity occurrence, (ii) cushion-shaped haustorial plate formation, (iii) haustorial (endophytic) primordium initiation in the cortex, with growth penetrating into the stem of the host, and finally (iv) tracheary element differentiation and connection with host’s vessels. Phloem sieve elements differentiation was not observed in this association. *C. filiformis* had developed xylem and degenerating phloem, which suggested mainly water and inorganic nutrition absorption of *C. filiformis* from the hosts such as *S. purpurea* (Li and Yao, 1992). It has been noted that the lack of phloem connections in haustoria with host plants is one of the substantial differences between the stem parasitic vines *Cassytha* and *Cuscuta* (Těšitel, 2016). However, in the parasitic interaction between *C. filiformis* and *M. tinctoria*, it has been reported that *Cassytha* haustoria have made contact with the phloem to obtain photosynthetic nutrients (Balasubramanian et al., 2014). The difference may be due to different host species or different infection stages, which needs well-coordinated further studies.

In addition to both mechanical and physical activities involved with the successful haustorial establishment of *Cassytha* with hosts, biochemical processes are also involved. The combination of these processes facilitates the quick, successful attachment of *Cassytha* haustoria to the conducting tissues of hosts. The penetrating haustoria of *C. filiformis* can release acid phosphatase (ACP) to injure host cells in conjunction with mechanical breakage of host cortical cells (Yao et al., 1994). Additionally, when twining on the host *S. purpurea*, the starch granules in *C. filiformis* stems have been seen to increase near the host end and further accumulated in the cells of critical regions along with haustorial development. After penetrating the host, starch granules gradually decrease and then disappear in the haustorium. The allocation and change of protein were contrary to that of the starch granules, indicating that when and where the starch granules decrease, the protein content increases and vice versa (Li and Yao, 1992). These results indicate that starch hydrolysis and protein synthesis provide matter and energy for cell division and other biochemical activities during haustorial development. Yao et al. (1994) have further suggested that haustorial development is closely correlated with the hormone cytokinin (CTK). The evidence for the above statement was found when haustoria of *C. filiformis* attached to the host *Salix integra*, and the isopentenyl adenine (iPa) and zeatin nucleotide (ZR) contents in the haustorial primordium initiation stage were observed to be much higher than those of the twining stage and penetrating stage.

### Distribution and Host Range of *Cassytha*

**FIGURE 2:**
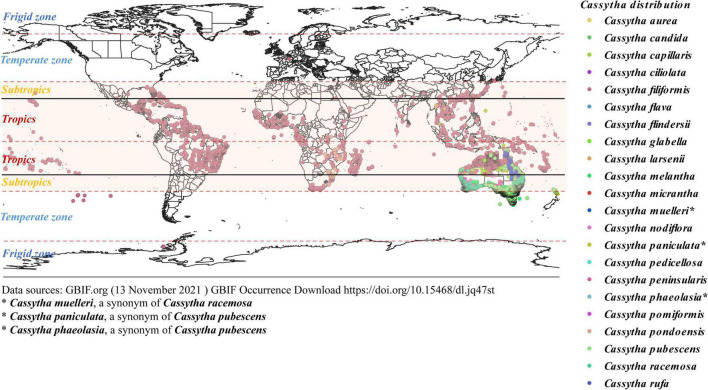
Global distribution map of *Cassytha* species across climatic zones. We modified the GBIF map according to published literature and POWO to indicate the world distribution of *Cassytha*.

*Cassytha* species are mainly distributed in tropics and subtropical regions (Figure 2), in coastal habitats and some species also in shrublands and forests (Table 1; Olson and Dinerstein, 2002). *Cassytha* is considered to be shade intolerant and is found to be best developed on relatively shorter trees and shrubs in open habitats, especially by roadsides and coastal vegetation (Werth et al., 1979). *Cassytha* is reported to parasitize a wide range of herbaceous and woody host species. But a survey conducted by Buriyo et al. (2015) in a cashew growing area in Tanzania has reported that *C. filiformis* has parasitized 75.4% of tree species followed by 23.2% of shrub species. In sharp contrast, herbaceous plants were rarely parasitized. We summarized 272 affirmatory host species of six *Cassytha* species from published literature covering 10 countries or regions, among which 226 are woody plants (Supplementary Appendix Table S2). This preference may accord with the perennial life form and hemiparasitic nature of *C. filiformis*. Herbaceous species might be bridging hosts that allow juvenile *Cassytha* to grow toward the perennial shrub or tree hosts in shrublands or forest ecosystems.

According to the species list we collected, Fabaceae is the most preferred host family parasitized by *Cassytha* based on species number, followed by family Myrtaceae and Asteraceae (Table 2). There are 32 reported host species in the Fabaceae family from 7 countries and regions, 19 host species for Myrtaceae, and 16 host species for Asteraceae. In this respect, *Acacia* (six host species in this genus) and *Eucalyptus* (five species in this genus) are the most preferred host genus parasitized by *Cassytha* (Supplementary Appendix Table S2). For example, *Acacia auriculiformis, Acacia confusa*, and *Acacia sieberiana* are recognized to be host species of *C. filiformis* in Benin (Quetin-Leclercq et al., 2004), China (Gong, 1986), and Tanzania (Buriyo et al., 2015), respectively. The Key to Tasmanian Vascular Plants (2019) states that *C. melantha* is distributed across a range of woody species and *Acacia* spp., such as *Acacia melanoxylon* is recognized as its preferred host plant (Ziegler, 1995; Dueholm et al., 2017). *Acacia myrtifolia* and *Acacia paradoxa* are also preferred host species for *C. pubescens* (Cirocco et al., 2017; Facelli et al., 2020). *Eucalyptus tetrodonta* is the host species for *C. filiformis* in Australia (Ziegler, 1995), and *Eucalyptus citriodora, Eucalyptus exserta, Eucalyptus robusta*, and *Eucalyptus rudis* are host species for *C. filiformis* in China (Gong, 1986; Li et al., 1992). *C. melantha* has caused severe damage to *Eucalyptus* spp. in Australia (Pederick and Zimmer, 1961) and Eucalypts are found to be specific hosts for *C. melantha* in Western Australia (Archer, 2012). Additionally, *C. filiformis* also infect crop species such as cashew (*Anacardium occidentale*), orange (*Citrus sinensis*), lemon (*Citrus limon*), mango (*Mangifera indica*), cloves (*Eugenia aromatica*), nutmeg (*Myristica fragrans*), and avocado (*Persea americana*) (Nelson, 2008; Buriyo et al., 2015).

**TABLE 2:** Host plant family susceptible to *Cassytha* infestation all over the world.

Order	Host family	Number of host genus	Number of host species	Countries or regions	Parasitic *Cassytha* species
1	Acanthaceae	1	1	India	*C. filiformis*
2	Altingiaceae	1	1	China	*C. filiformis*
3	Anacardiaceae	6	9	Benin, China, Tanzania, United States	*C. filiformis*
4	Annonaceae	1	1	China	*C. filiformis*
5	Apiaceae	1	1	Japan	*C. filiformis*
6	Apocynaceae	6	4	China, India, Pakistan	*C. filiformis*
7	Aquifoliaceae	1	1	China	*C. filiformis*
8	Araliaceae	1	1	China	*C. filiformis*
9	Arecaceae	1	2	India	*C. filiformis*
10	Asphodelaceae	1	1	China	*C. filiformis*
11	Aspleniaceae	1	1	China	*C. filiformis*
12	Asteraceae	15	16	China, India, Japan, Tanzania	*C. filiformis*
13	Bignoniaceae	1	1	China	*C. filiformis*
14	Boraginaceae	1	1	China, Hawaii	*C. filiformis*
15	Casuarinaceae	1	2	Australia, China, Japan	*C. filiformis, C. glabella*
16	Celastraceae	1	1	Japan	*C. filiformis*
17	Combretaceae	3	3	China, India, Tanzania	*C. filiformis*
18	Convolvulaceae	1	2	China, Japan	*C. filiformis*
19	Cornaceae	1	1	India	*C. filiformis*
20	Cupressaceae	1	1	China	*C. filiformis*
21	Cyperaceae	1	1	Japan	*C. glabella*
22	Daphniphyllaceae	1	1	China	*C. filiformis*
23	Dioscoreaceae	1	1	India	*C. filiformis*
24	Dipterocarpaceae	1	1	India	*C. filiformis*
25	Ebenaceae	1	1	India	*C. filiformis*
26	Elaeocarpaceae	1	1	China	*C. filiformis*
27	Euphorbiaceae	9	13	China, India, Japan, Tanzania	*C. filiformis*
28	Fabaceae	21	32	Australia, Benin, China, India, Japan, Pakistan, Tanzania	*C. filiformis, C. melantha, C. pubescens*
29	Fagaceae	3	3	China	*C. filiformis*
30	Gelsemiaceae	1	1	China	*C. filiformis*
31	Gleicheniaceae	1	1	China, Japan	*C. filiformis*
32	Goodeniaceae	1	2	Hawaii, Japan	*C. filiformis*
33	Hamamelidaceae	1	1	China	*C. filiformis*
34	Hypericaceae	1	1	China	*C. filiformis*
35	Juglandaceae	1	1	China	*C. filiformis*
36	Lamiaceae	4	5	Benin, China, Tanzania	*C. filiformis*
37	Lauraceae	5	9	China, Japan	*C. filiformis*
38	Liliaceae	1	1	India	*C. filiformis*
39	Lythraceae	1	1	Tanzania	*C. filiformis*
40	Magnoliaceae	1	1	China	*C. filiformis*
41	Malvaceae	8	12	China, Denmark, India, Tanzania	*C. filiformis, C. pubescens*
42	Melastomataceae	1	1	China	*C. filiformis*
43	Meliaceae	4	4	China, Benin, Tanzania	*C. filiformis*
44	Menispermaceae	1	2	China, India	*C. filiformis*
45	Moraceae	6	6	China, India, Pakistan, Tanzania	*C. filiformis*
46	Myristicaceae	1	1	Not available	*C. filiformis*
47	Myrtaceae	13	19	Australia, China, Hawaii, India, Tanzania	*C. filiformis, C. flava, C. glabella, C. melantha, C. pomiformis, C. pubescens*
48	Nyctaginaceae	1	1	Pakistan	*C. filiformis*
49	Ochnaceae	1	1	Tanzania	*C. filiformis*
50	Oleaceae	1	1	China	*C. filiformis*
51	Pandanaceae	1	1	Hawaii, Japan	*C. filiformis*
52	Phyllanthaceae	7	11	China, India, Tanzania	*C. filiformis*
53	Pinaceae	2	2	China	*C. filiformis*
54	Poaceae	10	10	China, Japan, Tanzania	*C. filiformis, C. glabella*
55	Primulaceae	3	3	China, Japan	*C. filiformis*
56	Proteaceae	1	1	Australia	*C. glabella*
57	Pteridaceae	2	2	China	*C. filiformis*
58	Ranunculaceae	1	1	China	*C. filiformis*
59	Rhamnaceae	5	8	China, India, Pakistan	*C. filiformis*
60	Rosaceae	1	1	China	*C. filiformis*
61	Rubiaceae	7	12	China, Hawaii, India, Tanzania	*C. filiformis*
62	Rutaceae	5	7	China, Japan, Pakistan, Tanzania	*C. filiformis*
63	Salicaceae	4	6	China, India	*C. filiformis*
64	Sapindaceae	4	4	China, India, Japan	*C. filiformis*
65	Sapotaceae	2	2	China, India	*C. filiformis*
66	Simaroubaceae	1	1	China	*C. filiformis*
67	Smilacaceae	1	1	Japan	*C. filiformis*
68	Solanaceae	1	1	China	*C. filiformis*
69	Styracaceae	1	1	China	*C. filiformis*
70	Symplocaceae	1	2	China	*C. filiformis*
71	Theaceae	3	8	China	*C. filiformis*
72	Thymelaeaceae	1	1	China	*C. filiformis*
73	Ulmaceae	1	1	India, Tanzania	*C. filiformis*
74	Verbenaceae	3	4	China, India, Japan, Tanzania	*C. filiformis*
75	Viburnaceae	1	1	China	*C. filiformis*
76	Vitaceae	2	2	China, India	*C. filiformis*

Twenty-eight host species of *C. filiformis* from India (Nayar and Nayar, 1952) and 81 host species from the Bahamas (Werth et al., 1979) are mentioned but have not been accompanied by a detailed list. *C. filiformis* was also reported to be found in Brazil (Giannerini et al., 2015), Nigeria (Ambi et al., 2017), Puerto Rico (Levins and Heatwole, 1973), Polynesia, Sri Lanka, Bangladesh (Ara et al., 2007), Brunei Darussalam (Tennakoon et al., 2016), Vietnam, Malaysia, Philippines, Indonesia, and Fiji (Nugraha et al., 2020), but information regarding host species is lacking. *C. glabella* is found to be a climber of many plant communities in Gibraltar Range and part of Washpool National Parks in New South Wales, Australia, including *Eucalyptus olida*–*Eucalyptus ligustrina*–*Eucalyptus cameronii* forest and woodland, *Baeckea omissa*–*Epacris obtusifolia*–*Leptospermum arachnoides* bogs, and *Callicoma serratifolia*–*Eucalyptus oreades* open forest and shrubland (Hunter and Sheringham, 2008). *C. ciliolata* is known as a common parasite in the Cape region of South Africa with a number of hosts (Schroeder, 1967). Unfortunately, this study has not reported the specific host plants parasitized by *C. ciliolata*. *Cassytha pedicellosa*, endemic to Tasmania, is distributed in heathland habitat and its associated species include *Lepidosperma concavum, Leptospermum scoparium, Hibbertia procumbens, Banksia marginata, Dillwynia glaberrima, Amperea xiphoclada, Epacris impressa, Monotoca scoparia, Monotoca glauca, Allocasuarina monilifera*, and *Selaginella uliginosa* (Wapstra et al., 2009). However, it is not explicitly stated if these are true host species (establishing successful haustorial connections) of *C. pedicellosa*. More host species for *Cassytha* should be identified in the *Cassytha* distribution habitats, so as to confirm their host preference.

Despite the availability of a wide range of host species, the level of infection by *Cassytha* varies among those hosts. For example, 30 host species in forests of the Jhargram district of West Bengal had 30–98% infection percentage/frequency parasitized by *C. filiformis* (Debabrata, 2018). Werth et al. (1979) have stated that the 81 host species from the Bahamas were not equally infected. Additionally, it has been reported that *C. filiformis* has a broader host range than *Cuscuta* in Brunei Darussalam, but the host preference (true haustorial initiation) is much narrower (Tennakoon et al., 2016). In line with our expectation, *Cassytha* tended to parasitize woody host species and species from certain families. *Cassytha* may have a variable preference for host species, perhaps due to the availability of more suitable host-derived resources in those plants. Different host species may also have different susceptibility, i.e., resistance levels to *Cassytha* parasitism. It is not known what factors might contribute to the susceptibility of various hosts. One study direction may be to investigate host stem exogenous histology and *Cassytha* haustorial penetration behavior. Host plants with soft thin barks and periderm seem to be more preferred by *C. filiformis* than species having hard-thick or suberized-scaly barks (Buriyo et al., 2015). Another study direction would be to compare the growth habits of host species, such as height and branch quantity. For example, *C. filiformis* seems to prefer low and much-branched woody host plants (Werth et al., 1979).

## Influence of *Cassytha* Parasite on Host Growth and Development

In this section, we discuss the impact of *Cassytha* parasitism on ecological, physiological, and molecular aspects of host species.

### Effect on Growth and Photosynthesis

**FIGURE 3:**
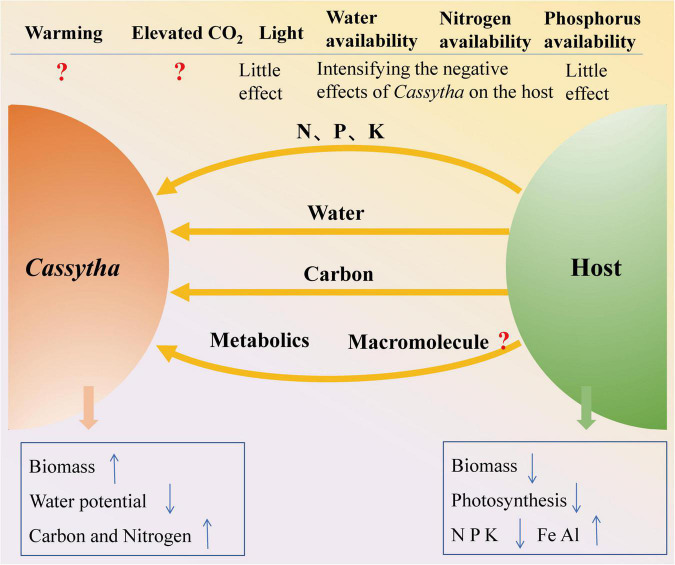
Frame diagram of *Cassytha*–host interactions and the impacts of environmental change.

*Cassytha* usually absorbs xylem-derived nutrients and water from host plants, decreasing their growth, reproduction, and biomass (Figure 3) and can even lead to the death of some hosts under heavy infestation (Burch, 1997; Prider et al., 2011). It has been reported that *C. pubescens* reduced the flowering of the legume host *C. scoparius* by 50% and consequently impacted fruit and seed production (Prider et al., 2011). Additionally, the noxious alien invasive weed *U. europaeus* when parasitized by native *C. pubescens* in South Australia, had a significantly lower shoot, root, and total biomass. The total biomass of infected hosts was 65–88% lower than the of uninfected plants (Cirocco et al., 2020). The adverse impact of the parasite on small invasive shrub host plants *U. europaeus* was more severe than on larger plants within the same species. On the other hand, the biomass of the parasite was lower when it was parasitizing smaller host plants, but was similar on a per gram of host total biomass basis in *C. pubescens* (Cirocco et al., 2020). This pattern may be expected at the cross-species level because Cirocco et al. (2021a) suggested that the native host was strongly affected by *C. pubescens* due to its smaller size. We do not know if this is true for other *Cassytha*–host associations involving different *Cassytha* species and/or other host species.

Biomass decrease of host species is partially attributed to decreased photosynthesis by *Cassytha* parasitism. It has been found that the photosynthetic rates, stomatal conductance, transpiration rate, light-saturated electron transport rates, pre-dawn (*F*_*v*_/*F*_*m*_), and midday (Φ_*PSII*_) quantum yields of the *C. pubescens* parasitized host *C. scoparius* were significantly lower than uninfected plants (Shen et al., 2010). It has also been reported that *Cassytha* infection significantly decreases midday PSII efficiency and the maximum electron transport rates of the alien invasive shrub host *U. europaeus*, regardless of different environmental variations across several field sites in South Australia (Cirocco et al., 2018). The results may be correlated to the decreased N and K levels in infected plants due to *Cassytha* infestation and increased Fe and Al content due to rhizosphere acidification induced by parasitism. This inevitably leads to suppressed photosynthesis and ultimately to chronic photoinhibition (Cirocco et al., 2018).

### Water and Nutrient Transmissions

Water and nutrient transmissions are key issues leading to an understanding of parasite–host interactions (Bell and Adams, 2011). The essential feature of water movement from soil to plants, and from host species to parasites, is a gradient of decreasing water potential (Ehleringer and Marshall, 1995). The water potential is usually more negative and stomatal conductance and transpiration rate are greater for aerial hemiparasites than for host species (Ehleringer and Marshall, 1995). However, water relations of *Cassytha*–host associations are generally sparse and have produced inconsistent results. *Cassytha* parasitism has been found to have no effect (Cirocco et al., 2016a, 2020) or negative effects (Cirocco et al., 2021b) on the water potential of the alien host *U. europaeus*. It has been further reported that the parasite’s water potential was significantly lower than the host, and the parasite had significantly lower water potential under low water than high water conditions (Cirocco et al., 2021b). Additionally, stomatal conductance and transpiration rates of the host *C. scoparius* infected by *C. pubescens* were significantly decreased when compared with uninfected plants (Shen et al., 2010).

Some angiosperm parasites can become a sink for host-produced photosynthates (Watling and Press, 2001). The facultative root parasite *Rhinanthus minor*, the obligate root hemiparasites *Striga* spp. and the root holoparasite *Orobanche* spp. obtain approximately 10, 30, and 100% of the carbon requirements from their hosts, respectively (Irving and Cameron, 2009). Stem hemiparasitic mistletoes have obtained around 40–80% heterotrophic carbon from hosts and it has been reported that the proportion of heterotrophic carbon gained by mistletoes depends on different host species and life history stages (Těšitel et al., 2010). The proportion of heterotrophic carbon obtained by *Cassytha* species, which subsists on xylem-derived solutes such as amino acids, sugars, and organic acids is not known. This is an area that requires further studies to understand the physiological implications of *Cassytha* infestation on different hosts.

Parasite resource removal from the host may be the primary mechanism for decreases in host biomass (Cirocco et al., 2021a). *Cassytha* primarily absorbs nutrients *via* xylem-xylem contact with the host species (Li and Yao, 1992). Studies conducted in South Australia have shown that N and K contents in infected plants of the alien invasive leguminous host *U. europaeus* by the parasite *C. pubescens* decreased by 17.6 and 22.4% compared with the uninfected plants, but the Al and Fe contents increased 140.5 and 40.5% due to rhizosphere acidification induced by parasitism (Cirocco et al., 2018). Additionally, the N, P, and K concentration of the parasite *C. pubescens* is higher when infecting small host plants of *U. europaeus* than in large ones (Cirocco et al., 2020).

### Metabolites and Molecules Translocation

Metabolites usually change and translocate between parasites and host plants prior to and after parasitism, usually from the former to the latter (Birschwilks et al., 2007). The energy charge calculated from ATP (adenosine triphosphate), ADP (adenosine diphosphate), and AMP (adenosine monophosphate) of *C. filiformis* seedlings were low prior to parasitism and greatly increased after parasitizing *Ipomoea pes-caprae* due to effective energy production, thus resulting in further elongation and development of *Cassytha* seedlings (Furuhashi et al., 2016). However, the energy charge of the host species *I. pes-caprae* did not change due to parasitism, which indicates that *I. pes-caprae* was relatively tolerant to water and metabolites loss to *Cassytha*. The profiled steroid pattern was not affected by parasitism in both *C. filiformis* and *I. pes-caprae*, but the absolute abundance of these steroids tended to decrease after parasitism. Similarly, the absolute abundance of most polar metabolites (such as fructose, glucose, sucrose, and galactitol) of *C. filiformis* decreased after parasitism thus attributing to water absorption from the host and the lignification process that induces fresh weight increase. However, for the host *I. pes-caprae*, the absolute amount of fructose, glucose, and sucrose decreased, whilst that of galactitol increased and pinitol, quinate, and organic acids did not change after *Cassytha* parasitism (Furuhashi et al., 2016). These results indicate that parasitism did not cause severe pathogenic responses in *I. pes-caprae*, although the growth and reproductive traits were negatively affected. It is not known if these patterns can be generalized for other *Cassytha*–host associations. Further studies are called for in this discipline. *Cassytha* can also absorb and accumulate secondary metabolites from host species. For example, gelsemium alkaloids were detected in *C. filiformis* when it was grown in association with the poisonous “heartbreak grass” *Gelsemium elegans* and absorbed the gelsemium toxins present in cell sap (Cheung et al., 2018). This implies the importance of assessing the hosts parasitized by *Cassytha* when they are harvested for medicinal preparations.

Parasite plants acquire various macromolecules such as mRNA, viruses, protein, and phytoplasmas from their hosts (LeBlanc et al., 2012). Some reports are available for parasites such as *Cuscuta, Cytinus*, Convolvulaceae, and Santalales (LeBlanc et al., 2012) showing them acquiring macromolecules from their hosts. For example, *Cuscuta* has the ability to transmit viruses and phytoplasmas between different hosts as a vector, being commonly called a *Cuscuta* “bridge” (Marcone et al., 1997; Birschwilks et al., 2006). Horizontal gene transfer is another example of parasite–host macromolecule exchange. We do not know if *Cassytha* can acquire viruses or other macromolecules from their hosts, but some evidence is available to demonstrate *Cassytha*–host associations involving horizontal gene transfer (Davis and Xi, 2015).

### Comparison of *Cassytha* Infection on Different Hosts

Host plants may show different resistance/tolerance levels to *Cassytha* parasitism. In a study conducted by Facelli et al. (2020), the native *Cassytha* is shown to have greater impacts on their exotic hosts than the native host plants in South Australia. There have been several case studies done in Australia under both field and glasshouse conditions to assess the impacts of *Cassytha* in this regard. For example, *C. pubescens* infection had a significant negative effect on the transpiration rates and biomass production of the alien invasive shrub host *C. scoparius* compared with that of native shrub host *Leptospermum myrsinoides.* Intense *C. pubescens* infection can even induce death for the host *C. scoparius* (Prider et al., 2009). *C. pubescens* parasitism has also been shown to significantly decrease the total biomass of the alien invasive host *U. europaeus*, but not the native host *A. paradoxa* (Cirocco et al., 2017). In addition, *C. pubescens* had higher photosynthetic rates, growth rates and biomass when parasitizing introduced hosts than the native hosts (Prider et al., 2009). These variations may be attributed to the greater level of resources (as higher nutrient contents) that the introduced host can provide and/or the greater resistance made by the native hosts against the successful *Cassytha* haustorial establishment. In order to understand the level of resistance exhibited by different hosts, Facelli et al. (2020) investigated the flow of nutrients between hosts and parasites. It was found that the connections of the haustorium with the vascular system of the native host *A. myrtifolia* were not successfully developed despite being morphologically alike to those formed on the alien invasive hosts *C. scoparius* and *U. europaeus*. They further demonstrated that radiolabeled phosphorus (^32^P) was not transferred from the native host *A. myrtifolia* to the parasite *C. pubescens* due to the incompatibility of haustorial connections. No definitive studies are available on the resistance exhibited by different hosts toward *Cassytha* infection.

## Impacts of Environmental Change on *Cassytha*–Host Interactions

In this section, we aim to test whether global environmental changes favor the hemiparasitic *Cassytha* or their hosts, specifically under elevated temperature and CO_2_ concentrations, and fluctuating water and soil nutrient conditions.

### Impacts of Temperature and Elevated CO_2_ Levels on *Cassytha*–Host Interactions

Both biotic factors and abiotic factors can alter parasite performance and its impact on host species, leading to compounded parasite–host behavior. Temperature is the prevailing environmental factor that influences plant growth, and also affects angiosperm parasite–host interactions. A case study involving the interaction between the hemiparasite *Castilleja sulphurea* and its host *Bouteloua gracilis* under circumstances of changing environment has found that a 3°C temperature increase in summer exacerbated the adverse effects on host species due to the production of more haustoria and aboveground biomass of the hemiparasite (Rafferty et al., 2019). Bell et al. (2020) have also found increased effects of the dwarf mistletoe (*Arceuthobium tsugense*) on hemlock *Tsuga heterophylla* under warmer and drier conditions. Similarly, the proportion of mistletoe *Viscum album* infection on *Pinus nigra* and *Pinus sylvestris* declined with the elevational increase (viz. temperature decrease) (Zamora and Mellado, 2019). The frequency of *C. pubescens* in Mediterranean climate healthy woodlands in South Australia has decreased from 1986 to 2010 due to a mean temperature increase of 4°C in those habitats (Guerin and Lowe, 2013). However, the exact impacts of temperature fluctuations on the overall dynamics of *Cassytha*–host associations are yet unknown.

Elevated CO_2_ alleviated the effects of the root holoparasite *Orobanche minor* on host species *Trifolium repens* by stimulating the host growth (Dale and Press, 1998). Similar results were found in the facultative hemiparasite *R. minor* and its host *Poa pratensis*, the root hemiparasitic angiosperm *Striga hermonthica*, and its host *Oryza sativa* and the aerial hemiparasitic plant *Dendrophthoe curvata* and its host species *Andira inermis, M. indica*, and *Vitex pinnata* under elevated CO_2_ (Watling and Press, 2000; Hwangbo et al., 2003; Le et al., 2016). No studies are reported about the influence of elevated CO_2_ on *Cassytha*–host associations.

### Impacts of Water Availability on *Cassytha*–Host Interactions

Water availability is another important environmental factor that influences angiosperm parasite–host plant interactions. Drought has been shown to decrease hosts’ growth rate and resource availability, thus indirectly influencing parasites (Zagorchev et al., 2021). It has been shown that the success of mistletoe establishment is related to host water status and the proportion of mistletoe infection decreased with the increase of water stress experienced by hosts (Miller et al., 2003). Cirocco et al. (2016a) have found that high water availability increased the negative effects of *C. pubescens* when parasitizing *U. europaeus*, with significantly lower host total biomass and parasite grew better at high water availability than in low water availability conditions. The predawn PSII efficiency of *U. europaeus* parasitized by *C. pubescens* was relatively low in wettest sites than in drier habitats (Cirocco et al., 2018). The physiological basis of this result is that the parasite *C. pubescens* had higher water potential, stomatal conductance, and growth rate at high water availability, leading to a higher demand of host-derived resources from the host *U. europaeus*, thus making it perform rather poorly.

### Light Effects on *Cassytha*–Host Interactions

The angiosperm aerial parasites decrease the photosynthesis of host species (Bell and Adams, 2011) and affect host PSII efficiency and the use of available light (Cameron et al., 2008). It has been shown that the parasite *C. pubescens* significantly decreased the foliar pigment concentration of the host species *L. myrsinoides* under both high and low photosynthetically active radiation (PAR) levels (Cirocco et al., 2015). However, infected *L. myrsinoides* plants have also maintained a similar photoprotective capacity similar to those uninfected plants irrespective of exposure to different light levels, thus preventing photodamage and demonstrating tolerance to *Cassytha* parasitism. Additionally, it has been found that a larger parasite growing on a larger host in high light had the same negative effect on host growth as a smaller parasite growing on a smaller host in low light (Cirocco et al., 2016b). Further in-depth studies are required to exactly understand the influence of different light conditions (e.g., light intensity and quality) on the overall behavior of *Cassytha*–host associations.

### Effects of Nitrogen and Phosphorus Availability on *Cassytha*-Host Interactions

Global atmospheric nitrogen (N) deposition is increasing due to human activities (Kanakidou et al., 2016), and these N effects on angiosperm parasite–host associations have long been recognized. A high external N supply has been found to reduce the effects of holoparasite on host growth due to less influence of infection on root biomass of the hosts (Shen et al., 2013) and negative effect on the early growth of parasite (Cechin and Press, 1993) compared with low external N supply. However, such influences were found to be different in *Cassytha*–native legume host and *Cassytha*–introduced legume host associations (Cirocco et al., 2017). High external N supply reduced the negative effects of *C. pubescens* infection on root biomass of the native legume species *A. paradoxa*, but it significantly increased the negative effects of *C. pubescens* infection on root biomass of the introduced legume species *U. europaeus*, compared with low external N supply. This is attributed to the reduction of nodule biomass of infected *U. europaeus* at a high external N supply when compared with the native host *A. paradoxa*. When native and introduced legume hosts are not parasitized by *C. pubescens*, external N supply had insignificant effects on their biomass (Cirocco et al., 2017). These results together with higher foliar N concentration present in native *A. paradoxa* than the exotic host *U. europaeus* indicate that the native host has adapted well by fixing more N to supply both its own and *Cassytha* growth. Other physiological aspects such as the photosynthetic efficiency of *Cassytha* infected hosts under N supplements, especially of that non-nitrogen fixing hosts are yet unknown. Cirocco et al. (2021b) further investigated the combined effects of water and nitrogen availability on *C. pubescens*–*U. europaeus* association, but did not find additive or antagonistic effects of water and nitrogen on parasite–host interaction. However, it was found that *C. pubescens* can absorb more nitrogen from the host *U. europaeus* at high water availability conditions.

Phosphorus is another essential nutrient that limits plant growth, which can influence angiosperm parasite–host associations (Davies and Graves, 2000). Cirocco et al. (2021a) conducted an experiment to assess the effects of external P supply on *C. pubescens* and a native legume *A. paradoxa* association. They found that external high P supply did not significantly influence the biomass of both the parasite *C. pubescens* and the host *A. paradoxa* compared with low P supply. However, the host *A. paradoxa* had significant lower foliar N and P concentration under low external P supply than high P supplements, resulting in lower stem phosphorus of *C. pubescens* in low external P supply than in high P supply treatment. The authors concluded that soil P conditions may have little or no impact on the overall performance of *Cassytha*–host associations in nature (Cirocco et al., 2021a). However, it may evidence three possibilities; first, different host species have divergent P sensitivities. Thus, the result may depend on different parasite–host combinations. Second, a 3-month experimental period is not long enough to detect P impact on the parasite–host association. Third, the effect of P supply on parasite-host association could be co-limited by N, because N and P are proportionally acquired by plants (Maistry et al., 2015). This topic clearly needs further study.

The major components of global environmental change are increasing CO_2_ concentrations, increasing temperatures, increasing N, and increasing or decreasing precipitation (Liu et al., 2017). Based on this literature review, the negative effects of the aerial hemiparasite *Cassytha* on their hosts increased under increasing water availability and N supply scenarios (Figure 3). Most previous studies on *Cassytha*–host associations have investigated the impacts of only one environmental factor. The intricacies involved with the cumulative impact of two or more of these factors on *Cassytha*–host associations have been investigated recently (e.g., Cirocco et al., 2021b) but still call for more research. A coordinated series of long-term studies are required to predict the performance of *Cassytha*–host associations under scenarios of climate change. It remains to be ascertained whether the presently documented evidence of the influence of factors such as temperature, water, sunlight, and nutrient (e.g., N and P) availability on *Cassytha*–host associations becomes more intense or mild until such complex studies are undertaken to assess the combined effects.

## Benefits and Harms of *Cassytha*–Host Interactions

In this section, we address the question of whether *Cassytha*–host interactions are generally beneficial or harmful for natural ecosystems and humans.

### Damage and Control of *Cassytha* as a Parasitic or Invasive Weed

Weeds act as significant biological constraints that can affect crop productivity (MacLaren et al., 2020). In this respect, the aerial hemiparasitic *Cassytha* species are a type of weed species (Musselman, 1996). For example, in tropical regions, parasite *C. filiformis* affect important economic crops such as *Acacia, Azadirachta, Mangifera*, Myrtaceae, and Theaceae (Li et al., 1991; Mythili et al., 2011). It has been found that 20% of cashew trees and 16% of orange trees were affected by *C. filiformis* in Tanzania, where 30–40% of total crop production is lost due to crop pests and diseases (Buriyo et al., 2015). The incidence of attacks on the forestry industry of southeastern China due to *C. filiformis* infestation exceeded 15%, reaching 50–60% in young *Camellia oleosa* forest in Guangxi Province (Gong, 1986).

Numerous parasitic plants including those of *Cassytha* have dramatic impacts on plant communities despite being less than 5% of the community biomass proportion, affecting community biomass, community diversity, vegetation cycling, and zonation aspects (Press and Phoenix, 2005). It has been found that *C. filiformis* invasion decreased the evenness and biomass but increased the density and species richness of aboveground plant communities in the forest of the Paracel Islands in the northern South China Sea (Cai et al., 2020). It also changed soil fauna and microbial community structure (Cai et al., 2020). *Cassytha* invasion may have both positive and negative influences on natural ecosystems and they might be keystone species. For example, *C. ciliolata* does well where there is a diverse range of hosts, e.g., in the Cunonia community of Cape floristic community (Meek et al., 2013).

Parasitic weed control is important for the protection of infected crops. In lightly infected regions, *C. filiformis* can be manually removed by hand-pulling, this being very efficient, especially at the seedling stage or when young stems are in the initial twining stages before producing flowers and fruits. In extensively infected regions, the application of suitable concentrations of selective herbicides such as Bentazon can be used to remove *C. filiformis* (Li et al., 1991). On the whole, invasive properties of *Cassytha* and their impacts on agricultural and natural communities and ecosystems need further study.

### Use of *Cassytha* as a Biocontrol Agent

Allelopathy is a biological phenomenon by which one plant can release chemicals that influence the survival and growth of plants in the same vicinity (Zhang et al., 2021). *C. filiformis* was shown to have negative allelopathic effects on three indicator plants *O. sativa, Echinochloa crus-galli* (Barnyardgrass), and *Vigna radiata*. Specific allelopathic effects of *Cassytha* on these plants were confirmed by applying *Cassytha* extracts in powder and water forms for bioassays, plant house studies, and field experiments (Thang et al., 2021). The dry weight of barnyardgrass was suppressed by 76.7 and 42.7% when *C. filiformis* extracts were applied in powder form in both net house and field trials. This study provided useful evidence about the potential of using *C. filiformis* as a natural herbicide to control weeds in non-paddy crop cultivation, but it is not known if other weed species can also be inhibited by *C. filiformis*.

Like *Cuscuta*, native *Cassytha* species can be used as a biocontrol agent to control plant invasion (Li et al., 2012; Těšitel et al., 2020). For example, the native parasite *C. filiformis* in Florida was recognized as a component of an integrated approach to managing the introduced and invasive tree *Schinus terebinthifolius. C. filiformis* combined with the leaflet rolling moth *Episimus unguiculus* herbivory greatly decreased the performance of *S. terebinthifolius* for at least 2 months after the removal of the moths (Manrique et al., 2009). The native hemiparasite *C. pubescens* in Australia also implied serious effects on the growth and biomass of the introduced legume species *U. europaeus* and *C. scoparius*, but not the native legume *A. paradoxa* and *L. myrsinoides* (Myrtaceae), under both glass house and field conditions (Prider et al., 2009; Cirocco et al., 2017). *C. pubescens* parasitism and seed predator *Bruchidius villosus* (Bruchidae) are found to have a sub-additive effect on the invasive species *C. scoparius*, which can be used as a good combination of biocontrol agents (Prider et al., 2011).

## Conclusion and Future Directions

*Cassytha* clearly demonstrates parasitism-related habits, such as flowering all year round for many species in this genus, autotrophy, stem twining, and producing haustoria. However, coordinated studies are required to precisely understand its seed biology and the duration of autotropism in order that its impact on the functioning of associated hosts in both agricultural and natural settings can be assessed. *Cassytha* tends to parasitize woody plants and species from certain families such as Fabaceae and Myrtaceae. However, it is not clear why *Cassytha* has varying levels of infection on different hosts. It may be due to factors such as less resources that a particular host provides, relatively high level of natural host resistance to parasitism, and incompatible host size or the anatomy of the host bark that can resist successful haustorial establishments. *Cassytha* absorbs water, N, P, and K nutrients, and possibly metabolites and macromolecules from host plants *via* haustoria to promote growth and increase its own biomass. While the growth, photosynthesis, reproduction, and biomass of some host plants were dramatically decreased. We are still far from understanding the underlying physiological and molecular level mechanisms of the extensive *Cassytha*–host interactions. For example, what roles do microorganisms play in parasitism of the *Cassytha*–host associations? Global environmental changes may increase the severity of *Cassytha* parasitism on host plants from increasing water and N availability perspectives. More studies are needed to ascertain the effects of multiple environmental factors on *Cassytha*–host associations, such as global warming, drought and N interactions and the influences of biological factors such as pollinators, predators, and microbes. *Cassytha* itself can be a harmful weed under heavy infestations, whilst it could be a biocontrol agent that can be used to reduce the spread of exotic weeds/invasive plants, and also a keystone species in natural ecosystems. Long-term community and ecosystem level field studies on *Cassytha*–host associations clearly need to be explored in a coordinated manner. Results of such studies would further improve our understanding of this aerial hemiparasite and will enable us to predict the trends of future spread of *Cassytha* under environmental change scenarios.

## Author Contributions

KT and SF developed the project. HZ and KT wrote the manuscript. SF reviewed the manuscript by rewriting, discussing, and commenting. All authors contributed to the manuscript and approved the submitted version.

## Conflict of Interest

The authors declare that the research was conducted in the absence of any commercial or financial relationships that could be construed as a potential conflict of interest.

## Publisher’s Note

All claims expressed in this article are solely those of the authors and do not necessarily represent those of their affiliated organizations, or those of the publisher, the editors and the reviewers. Any product that may be evaluated in this article, or claim that may be made by its manufacturer, is not guaranteed or endorsed by the publisher.

## Footnotes

1
http://www.theplantlist.org/

2
https://www.gbif.org/zh/

3
https://profiles.ala.org.au/opus/foa

4
https://biodiversity.org.au/nsl/services/search/taxonomy
