# Supplementary material for: The Angiosperm Stem Hemiparasitic Genus Cassytha (Lauraceae) and Its Host Interactions: A Review
Source: Front Plant Sci. 2022 Jun 6;13:864110. doi: 10.3389/fpls.2022.864110 (PMC9208266; doi:10.3389/fpls.2022.864110)
Supplement: Supplementary file 1 [file Table_1.docx]

**Appendix Table S1.** Changes for scientific names required for five *Cassytha* species according to the latest updates of the Plant List (accessed in 30 November, 2021).

|  | ***Cassytha* used as species in references** | **Distribution** | **References** | **Species in the Plant List** |
| --- | --- | --- | --- | --- |
| 1 | *Cassytha capensis* | Africa | Nickrent, 2020 | A synonym of ***Cassytha ciliolata*** |
| 2 | *Cassytha pergracilis* | Japan | Kokubugata et al., 2012, Kokubugata and Yokota, 2012 | A synonym of ***Cassytha glabella*** |
| 3 | *Cassytha muelleri* | Australia | Weber, 2007 | A synonym of ***Cassytha racemosa*** |
| 4 | *Cassytha paniculata* | Australia | Weber, 2007 | A synonym of ***Cassytha pubescens*** |
| 5 | *Cassytha phaeolasia* | Australia | Weber, 2007 | A synonym of ***Cassytha pubescens*** |

**Appendix Table S2 Host plants susceptible for *Cassytha* infestation all over the world**

| Cassytha **species** | **Countries or regions** | **Susceptible host species** | **Host family** | **Host life form** | **Reference** |
| --- | --- | --- | --- | --- | --- |
| *C. filiformis* | Benin | *Anacardium occidentale** | Anacardiaceae | Tree | Quetin-Leclercq et al., 2004 |
| *C. filiformis* | Australia | *Eucalyptus tetrodonta* | Myrtaceae | Tree | Ziegler, 1995 |
| *C. filiformis* | Benin | *Acacia auriculiformis* | Fabaceae | Tree | Quetin-Leclercq et al., 2004 |
| *C. filiformis* | Benin | *Mangifera indica** | Anacardiaceae | Tree | Quetin-Leclercq et al., 2004; Nelson, 2008 |
| *C. filiformis* | China | *Eupatorium odoratum** | Asteraceae | Herb | Li et al., 1992 |
| *C. filiformis* | Benin | *Hyptis suaveolens* | Lamiaceae | Herb | Quetin-Leclercq et al., 2004 |
| *C. filiformis* | Japan | *Bidens pilosa var. minor & var. radiata** | Asteraceae | Herb | Kokubugata and Yokota, 2012 |
| *C. filiformis* | China | *Acacia confusa* | Fabaceae | Tree | Gong, 1986 |
| *C. filiformis* | China | *Actinodaphne pilosa* | Lauraceae | Shrub or tree | Li et al., 1992 |
| *C. filiformis* | China | *Adiantum capillus-veneris* | Pteridaceae | Fern | Li et al., 1992 |
| *C. filiformis* | China | *Agibaria sinensis* | Thymelaeaceae | Tree | Li et al., 1992 |
| *C. filiformis* | China | *Antidesma bunius* | Phyllanthaceae | Tree | Li et al., 1992 |
| *C. filiformis* | China | *Antidesma ghaesembilla* | Phyllanthaceae | Shrub or tree | Li et al., 1992 |
| *C. filiformis* | China | *Aporosa chinensis* | Euphorbiaceae | Tree | Li et al., 1992 |
| *C. filiformis* | China | *Artemisia argyi* | Asteraceae | Herb | Li et al., 1992 |
| *C. filiformis* | China | *Asteromaea indica* | Asteraceae | Herb | Li et al., 1992 |
| *C. filiformis* | China | *Atropa belladonna* | Solanaceae | Herb | Li et al., 1992 |
| *C. filiformis* | China | *Baeckea frutescens* | Myrtaceae | Shrub or tree | Li et al., 1992 |
| *C. filiformis* | China | *Bambusa multiplex* | Poaceae | Herb | Li et al., 1992 |
| *C. filiformis* | China | *Berchemia lineata* | Rhamnaceae | Vine | Li et al., 1992 |
| *C. filiformis* | China | *Blainvillea acmella* | Asteraceae | Herb | Li et al., 1992 |
| *C. filiformis* | China | *Blechnum orientale* | Aspleniaceae | Fern | Li et al., 1992 |
| *C. filiformis* | China | *Breynia fruticosa* | Phyllanthaceae | Shrub or tree | Li et al., 1992 |
| *C. filiformis* | China | *Bridelia monoica* | Phyllanthaceae | Tree | Li et al., 1992 |
| *C. filiformis* | China | *Bridelia stipularia* | Phyllanthaceae | Vine | Li et al., 1992 |
| *C. filiformis* | China | *Brucea javanica* | Simaroubaceae | Shrub | Li et al., 1992 |
| *C. filiformis* | China | *Callistemon rigidus* | Myrtaceae | Shrub | Li et al., 1992 |
| *C. filiformis* | China | *Camcllia oleosa* | Theaceae | Shrub or tree | Gong, 1986 |
| *C. filiformis* | China | *Camellia oleifera* | Theaceae | Shrub or tree | Li et al., 1992 |
| *C. filiformis* | China | *Camellia vietnamensis* | Theaceae | Shrub or tree | Li et al., 1992 |
| *C. filiformis* | China | *Caryopteris incana* | Lamiaceae | Shrub | Li et al., 1992 |
| *C. filiformis* | China | *Castanea henryi* | Fagaceae | Tree | Li et al., 1992 |
| *C. filiformis* | China | *Castanopsis hystrix* | Fagaceae | Tree | Gong, 1986 |
| *C. filiformis* | China | *Tournefortia argentea** | Boraginaceae | Shrub or tree | Ren et al., 2017 |
| *C. filiformis* | China | *Cherodendrum cyrtophyllum* | Verbenaceae | Shrub | Li et al., 1992 |
| *C. filiformis* | China | *Chrysophyllum roxburghii* | Sapotaceae | Tree | Li et al., 1992 |
| *C. filiformis* | China | *Cinnamomum burmanni* | Lauraceae | Tree | Li et al., 1992 |
| *C. filiformis* | China | *Cinnamomum camphora* | Lauraceae | Tree | Gong, 1986; Li et al., 1992 |
| *C. filiformis* | China | *Cipadessa cinerascens* | Meliaceae | Shrub | Li et al., 1992 |
| *C. filiformis* | China | *Cirsium lineare* | Asteraceae | Herb | Li et al., 1992 |
| *C. filiformis* | China | *Citrus reticulata* | Rutaceae | Tree | Li et al., 1992 |
| *C. filiformis* | China | *Clematis armandi* | Ranunculaceae | Vine | Li et al., 1992 |
| *C. filiformis* | China | *Conyza viscidula* | Asteraceae | Herb | Li et al., 1992 |
| *C. filiformis* | China | *Cratoxylon ligustrinum* | Hypericaceae | Shrub or tree | Li et al., 1992 |
| *C. filiformis* | China | *Cryptolepsis buchanani* | Apocynaceae | Shrub | Li et al., 1992 |
| *C. filiformis* | China | *Cudrania tricuspidata* | Moraceae | Tree | Li et al., 1992 |
| *C. filiformis* | China | *Casuarina equisetifolia** | Casuarinaceae | Tree | Gong, 1986 |
| *C. filiformis* | China | *Cunninghamia lanceolata* | Cupressaceae | Tree | Gong, 1986 |
| *C. filiformis* | China | *Dalbergia obtusifolia* | Fabaceae | Tree | Li et al., 1992 |
| *C. filiformis* | China | *Dalbergia odorifera* | Fabaceae | Tree | Gong, 1986 |
| *C. filiformis* | China | *Daphniphyllum calycinum* | Daphniphyllaceae | Shrub | Li et al., 1992 |
| *C. filiformis* | China | *Dendranthema indicum* | Asteraceae | Herb | Li et al., 1992 |
| *C. filiformis* | China | *Dendrolobium triangulare* | Fabaceae | Shrub | Li et al., 1992 |
| *C. filiformis* | China | *Desmodium pulchellum* | Fabaceae | Shrub | Li et al., 1992 |
| *C. filiformis* | China | *Desmos cochinchinensis* | Annonaceae | Shrub or tree | Li et al., 1992 |
| *C. filiformis* | China | *Dianella ensibolia* | Asphodelaceae | Herb | Li et al., 1992 |
| *C. filiformis* | China | *Cunninghamia lanceolata** | Cupressaceae | Tree | Li et al., 1992 |
| *C. filiformis* | China | *Elaeocarpus sylvestris* | Elaeocarpaceae | Tree | Li et al., 1992 |
| *C. filiformis* | China | *Embelia henryi* | Primulaceae | Shrub | Li et al., 1992 |
| *C. filiformis* | China | *Engelhardtia colebrookiana* | Juglandaceae | Tree | Li et al., 1992 |
| *C. filiformis* | China | *Eucalyptus citriodora* | Myrtaceae | Tree | Gong, 1986 |
| ***C. melantha*** | Australia | *Acacia spp.** | Fabaceae | Tree | Dueholm et al., 2017 |
| *C. filiformis* | China | *Eucalyptus exserta* | Myrtaceae | Tree | Gong, 1986 |
| *C. filiformis* | China | *Eucalyptus robusta* | Myrtaceae | Tree | Li et al., 1992 |
| *C. filiformis* | China | *Eucalyptus rudis* | Myrtaceae | Tree | Gong, 1986 |
| *C. filiformis* | China | *Dicranopteris linearis** | Gleicheniaceae | Fern | Li et al., 1992 |
| *C. filiformis* | China | *Euphoria longan* | Sapindaceae | Tree | Li et al., 1992 |
| *C. filiformis* | China | *Eurya groffii* | Theaceae | Shrub or tree | Li et al., 1992 |
| *C. filiformis* | China | *Eurya nitida* | Theaceae | Shrub or tree | Li et al., 1992 |
| *C. filiformis* | China | *Eurya trichocarpa* | Theaceae | Shrub or tree | Li et al., 1992 |
| *C. filiformis* | China | *Evodia lepta* | Rutaceae | Shrub or tree | Li et al., 1992 |
| *C. filiformis* | China | *Flueggea virosa* | Phyllanthaceae | Shrub or tree | Li et al., 1992 |
| *C. filiformis* | China | *Gelsemium elegans* | Gelsemiaceae | Herb | Cheung et al., 2018 |
| *C. filiformis* | China | *Glochidion coccineum* | Phyllanthaceae | Shrub or tree | Li et al., 1992 |
| *C. filiformis* | China | *Glochidion fagifolium* | Phyllanthaceae | Shrub or tree | Li et al., 1992 |
| *C. filiformis* | China | *Glycosmis citrifolia* | Rutaceae | Shrub or tree | Li et al., 1992 |
| *C. filiformis* | China | *Gnaphalium multiceps* | Asteraceae | Herb | Li et al., 1992 |
| *C. filiformis* | China | *Gossampinus malabaricum* | Malvaceae | Tree | Li et al., 1992 |
| *C. filiformis* | China | *Grewia hirsuta* | Malvaceae | Shrub | Li et al., 1992 |
| *C. filiformis* | China | *Grewia tiliaefolia* | Malvaceae | Tree | Li et al., 1992 |
| *C. filiformis* | China | *Hedyotis auricularia* | Rubiaceae | Herb | Li et al., 1992 |
| *C. filiformis* | China | *Helicteres angustifolia* | Malvaceae | Shrub or tree | Li et al., 1992 |
| *C. filiformis* | China | *Helicteres glabriuscula* | Malvaceae | Shrub | Li et al., 1992 |
| *C. filiformis* | China | *Helicteres lanceolata* | Malvaceae | Shrub | Li et al., 1992 |
| *C. filiformis* | China | *Ilex asprella* | Aquifoliaceae | Shrub | Li et al., 1992 |
| *C. filiformis* | China | *Imperata cylinadrica* | Poaceae | Herb | Ho et al., 2004 |
| *C. filiformis* | China | *Inula cappa* | Asteraceae | Shrub | Li et al., 1992 |
| *C. filiformis* | China | *Ipomoea imperati* | Convolvulaceae | Herb | Ho et al., 2004 |
| *C. filiformis* | China | *Keteleeria fortunei* | Pinaceae | Tree | Li et al., 1992 |
| *C. filiformis* | China | *Khaya senegalensis* | Meliaceae | Tree | Gong, 1986 |
| *C. filiformis* | Benin | *Azadirachta indica** | Meliaceae | Tree | Quetin-Leclercq et al., 2004 |
| *C. filiformis* | China | *Lespedeza formosa* | Fabaceae | Shrub | Li et al., 1992 |
| *C. filiformis* | China | *Ligustrum sinense* | Oleaceae | Shrub | Li et al., 1992 |
| *C. filiformis* | China | *Lindera angustifolia* | Lauraceae | Shrub | Li et al., 1992 |
| *C. filiformis* | China | *Lindera strychnifolia* | Lauraceae | Shrub | Li et al., 1992 |
| *C. filiformis* | China | *Litsea cubeba* | Lauraceae | Shrub or tree | Li et al., 1992 |
| *C. filiformis* | China | *Liquidanmbar taiwaniana* | Altingiaceae | Tree | Li et al., 1992 |
| *C. filiformis* | China | *Litchi chinensis* | Sapindaceae | Tree | Li et al., 1992 |
| *C. filiformis* | China | *Litsea glutinosa* | Lauraceae | Tree | Li et al., 1992 |
| *C. filiformis* | China | *Maesa japonica* | Primulaceae | Shrub | Li et al., 1992 |
| *C. filiformis* | China | *Mallotus apelta* | Euphorbiaceae | Shrub or tree | Li et al., 1992 |
| *C. filiformis* | China | *Mallotus barbatus* | Euphorbiaceae | Shrub or tree | Li et al., 1992 |
| *C. filiformis* | China | *Mallotus philippinensis* | Euphorbiaceae | Tree | Li et al., 1992 |
| *C. filiformis* | China | *Mallotus repandus* | Euphorbiaceae | Vine | Li et al., 1992 |
| *C. filiformis* | China | *Melastoma candidum* | Melastomataceae | Shrub or tree | Li et al., 1992 |
| *C. filiformis* | China | *Melia azedarach* | Meliaceae | Tree | Gong, 1986; Li et al., 1992 |
| *C. filiformis* | China | *Michelia macclurei* | Magnoliaceae | Tree | Li et al., 1992 |
| *C. filiformis* | China | *Microstegium vagans* | Poaceae | Herb | Li et al., 1992 |
| *C. filiformis* | China | *Miscanthus floridulus* | Poaceae | Herb | Li et al., 1992 |
| *C. filiformis* | China | *Moghania macrophylla* | Fabaceae | Shrub | Li et al., 1992 |
| *C. filiformis* | China | *Mussaenda pubescens* | Rubiaceae | Shrub | Li et al., 1992 |
| *C. filiformis* | China | *Mytilaria laosensis* | Hamamelidaceae | Tree | Gong, 1986; Li et al., 1992 |
| *C. filiformis* | China | *Oldenlandia hedyotidea* | Rubiaceae | Shrub | Li et al., 1992 |
| *C. filiformis* | China | *Oroxylon indicum* | Bignoniaceae | Tree | Li et al., 1992 |
| *C. filiformis* | China | *Paliurus hemsleyanus* | Rhamnaceae | Shrub or tree | Li et al., 1992 |
| *C. filiformis* | China | *Paliurus ramosissmus* | Rhamnaceae | Shrub | Li et al., 1992 |
| *C. filiformis* | China | *Pesmodium triangulare#* | ? | ? | Li et al., 1992 |
| *C. filiformis* | China | *Phlomis umbrosa* | Lamiaceae | Herb | Li et al., 1992 |
| *C. filiformis* | China | *Phyllanthus emblica* | Phyllanthaceae | Tree | Li et al., 1992 |
| *C. filiformis* | China | *Pinus massoniana* | Pinaceae | Tree | Gong, 1986; Li et al., 1992 |
| *C. filiformis* | China | *Psidium guajava* | Myrtaceae | Shrub or tree | Li et al., 1992 |
| *C. filiformis* | China | *Psychotria rubra* | Rubiaceae | Tree | Li et al., 1992 |
| *C. filiformis* | China | *Pteria vittata* | Pteridaceae | Fern | Li et al., 1992 |
| *C. filiformis* | China | *Quercus fabri* | Fagaceae | Tree | Li et al., 1992 |
| *C. filiformis* | China | *Rhamnus crenata* | Rhamnaceae | Shrub | Li et al., 1992 |
| *C. filiformis* | China | *Rhodomyrtus tomentosa* | Myrtaceae | shrub | Li et al., 1992 |
| *C. filiformis* | China | *Rhus chinensis* | Anacardiaceae | Shrub or tree | Li et al., 1992 |
| *C. filiformis* | China | *Rhus silvestris* | Anacardiaceae | Tree | Li et al., 1992 |
| *C. filiformis* | China | *Rhus succedanea* | Anacardiaceae | Tree | Li et al., 1992 |
| *C. filiformis* | China | *Rubus cochinchinensis* | Rosaceae | Shrub | Li et al., 1992 |
| *C. filiformis* | China | *Sageretia theezans* | Rhamnaceae | Shrub | Li et al., 1992 |
| *C. filiformis* | China | *Salix integra* | Salicaceae | Shrub | Yao et al., 1994 |
| *C. filiformis* | China | *Salix purpurea* | Salicaceae | Shrub | Li and Yao, 1992 |
| *C. filiformis* | China | *Sapium discolor* | Euphorbiaceae | Tree | Li et al., 1992 |
| *C. filiformis* | China | *Sapium sebiferum* | Euphorbiaceae | Tree | Gong, 1986 |
| *C. filiformis* | China | *Schefflera octophylla* | Araliaceae | Tree | Li et al., 1992 |
| *C. filiformis* | China | *Schima superba* | Theaceae | Tree | Gong, 1986 |
| *C. filiformis* | China | *Schima wallichii* | Theaceae | Tree | Li et al., 1992 |
| *C. filiformis* | China | *Senecio erythropappus* | Asteraceae | Herb | Li et al., 1992 |
| *C. filiformis* | China | *Senecio scandens* | Asteraceae | Herb | Li et al., 1992 |
| *C. filiformis* | China | *Sindora tonkinensis* | Fabaceae | Tree | Li et al., 1992 |
| *C. filiformis* | China | *Stephania longa* | Menispermaceae | Herb | Li et al., 1992 |
| *C. filiformis* | China | *Streptocaulon griffithii* | Apocynaceae | Herb | Li et al., 1992 |
| *C. filiformis* | China | *Strophanthus divaricatus* | Apocynaceae | Shrub | Li et al., 1992 |
| *C. filiformis* | China | *Styrax faberi* | Styracaceae | Shrub | Li et al., 1992 |
| *C. filiformis* | China | *Symplocos confusa* | Symplocaceae | Tree | Li et al., 1992 |
| *C. filiformis* | China | *Symplocos racemosa* | Symplocaceae | Tree | Li et al., 1992 |
| ***C. filiformis*** | Australia | *Eucalyptus spp.** | Myrtaceae | Tree | Ziegler, 1995 |
| *C. filiformis* | China | *Syzygium jambos* | Myrtaceae | Tree | Li et al., 1992 |
| *C. filiformis* | China | *Terminalia hainanensis* | Combretaceae | Shrub or tree | Gong, 1986 |
| *C. filiformis* | China | *Toddalia asiatica* | Rutaceae | Vine | Li et al., 1992 |
| *C. filiformis* | China | *Eucalyptus exserta** | Myrtaceae | Tree | Li et al., 1992 |
| *C. filiformis* | China | *Uraria clarkei* | Fabaceae | Shrub | Li et al., 1992 |
| *C. filiformis* | China | *Urena lobata* | Malvaceae | Shrub | Li et al., 1992 |
| *C. filiformis* | China | *Vernicia fordii* | Euphorbiaceae | Tree | Li et al., 1992 |
| *C. filiformis* | China | *Viburnum fordiae* | Viburnaceae | Shrub | Li et al., 1992 |
| *C. filiformis* | China | *Vitex negundo* | Lamiaceae | Tree | Li et al., 1992 |
| *C. filiformis* | China | *Vitis pentagona* | Vitaceae | Vine | Li et al., 1992 |
| *C. filiformis* | China | *Wendlandia aberrans* | Rubiaceae | Shrub | Li et al., 1992 |
| *C. filiformis* | China | *Wendlandia pinpienensis* | Rubiaceae | Shrub or tree | Li et al., 1992 |
| *C. filiformis* | China | *Wendlandia uvariiolia* | Rubiaceae | Shrub or tree | Li et al., 1992 |
| *C. filiformis* | China | *Xylosma congestum* | Salicaceae | Shrub | Li et al., 1992 |
| *C. filiformis* | China | *Xylosma controversum* | Salicaceae | Shrub or tree | Li et al., 1992 |
| *C. filiformis* | Hawaii | *Metrosideros polymorpha* | Myrtaceae | Tree | Nelson, 2008 |
| *C. filiformis* | Hawaii | *Morinda citrifolia* | Rubiaceae | Tree | Nelson, 2008 |
| *C. filiformis* | China | *Syzygium cumini** | Myrtaceae | Tree | Gong, 1986; Li et al., 1992 |
| *C. filiformis* | Hawaii | *Scaevola sericea* | Goodeniaceae | Shrub | Nelson, 2008 |
| *C. filiformis* | Hawaii | *Tournefortia argentea* | Boraginaceae | Shrub or tree | Nelson, 2008 |
| *C. filiformis* | India | *Alangium lamarckii* | Cornaceae | Tree | Debabrata 2018 |
| *C. filiformis* | India | *Allophyllus cobbe* | Sapindaceae | Tree | Debabrata 2018 |
| *C. filiformis* | India | *Atylosia platycarpa* | Fabaceae | Herb | Debabrata 2018 |
| *C. filiformis* | India | *Bridelia retusa* | Euphorbiaceae | Tree | Debabrata 2018 |
| *C. filiformis* | India | *Bridelia scandens* | Phyllanthaceae | Vine | Rajanna and Shivamurthy, 2001 |
| *C. filiformis* | India | *Buettneria racemosa#* | Malvaceae | ? | Debabrata 2018 |
| *C. filiformis* | India | *Canthium parviflorum* | Rubiaceae | Shrub | Rajanna and Shivamurthy, 2001 |
| *C. filiformis* | India | *Canthium rheedii* | Rubiaceae | Shrub | Rajanna and Shivamurthy, 2001 |
| *C. filiformis* | India | *Casearia tomentosa* | Salicaceae | Tree | Debabrata 2018 |
| *C. filiformis* | India | *Cassia fistula* | Fabaceae | Tree | Debabrata 2018 |
| *C. filiformis* | India | *Cassia occidentalis* | Fabaceae | Shrub | Debabrata 2018 |
| *C. filiformis* | India | *Cassia tora* | Fabaceae | Herb | Debabrata 2018 |
| *C. filiformis* | India | *Cissus discolour* | Vitaceae | Vine | Debabrata 2018 |
| *C. filiformis* | India | *Combretum decundrum* | Combretaceae | Vine | Debabrata 2018 |
| *C. filiformis* | India | *Desmodium gangeticum* | Fabaceae | Shrub | Debabrata 2018 |
| *C. filiformis* | India | *Diciptera bupleuroides* | Acanthaceae | Herb | Debabrata 2018 |
| *C. filiformis* | India | *Dioscorea oppositifolia* | Dioscoreaceae | Vine | Rajanna and Shivamurthy, 2001 |
| *C. filiformis* | India | *Diospyros melnoxylon* | Ebenaceae | Tree | Debabrata 2018 |
| *C. filiformis* | India | *Eupatorium odoratum* | Asteraceae | Herb | Debabrata 2018 |
| *C. filiformis* | India | *Flacourtia indica* | Salicaceae | Shrub or tree | Debabrata 2018 |
| *C. filiformis* | India | *Flemingia chapper* | Fabaceae | Shrub | Debabrata 2018 |
| *C. filiformis* | India | *Gloriosa superb* | Liliaceae | Herb | Debabrata 2018 |
| *C. filiformis* | India | *Holarhena antidysenterica* | Apocynaceae | Shrub or tree | Debabrata 2018 |
| *C. glabella* | Australia | *Leptomeria scoparium** | Myrtaceae | Shrub | Close et al., 2006 |
| *C. filiformis* | India | *Madhuca indica* | Sapotaceae | Tree | Debabrata 2018 |
| *C. filiformis* | India | *Morinda tinctoria* | Rubiaceae | Shrub or tree | Balasubramanian et al., 2014 |
| *C. filiformis* | India | *Phoenix acaulis* | Arecaceae | Shrub | Debabrata 2018 |
| *C. filiformis* | India | *Phoenix sylvestris* | Arecaceae | Tree | Debabrata 2018 |
| *C. filiformis* | India | *Phyllanthus reticulatus* | Euphorbiaceae | Shrub | Debabrata 2018 |
| *C. filiformis* | India | *Shorea robusta* | Dipterocarpaceae | Tree | Debabrata 2018 |
| *C. filiformis* | India | *Stephania japonica* | Menispermaceae | Vine | Debabrata 2018 |
| *C. filiformis* | India | *Streblus asper* | Moraceae | Tree | Debabrata 2018 |
| *C. filiformis* | India | *Syzigium caryophyllaeum* | Myrtaceae | Tree | Rajanna and Shivamurthy, 2001 |
| *C. melantha* | Australia | *Eucalyptus spp.** | Myrtaceae | Tree | Pederick and Zimmer, 1961 |
| *C. filiformis* | India | *Ziziphus jujuba* | Rhamnaceae | Shrub or tree | Abubacker et al., 2005; Debabrata 2018 |
| *C. filiformis* | India | *Ziziphus oenoplea* | Rhamnaceae | Shrub | Debabrata 2018 |
| *C. filiformis* | Hawaii | *Pandanus* spp.* | Pandanaceae | Shrub or tree | Nelson, 2008 |
| *C. filiformis* | Japan | *Breynia vitis-idaea* | Euphorbiaceae | Shrub or tree | Kokubugata and Yokota, 2012 |
| *C. filiformis* | Japan | *Canavalia lineata* | Fabaceae | Vine | Kokubugata and Yokota, 2012 |
| *C. filiformis* | Japan | *Casuarina stricta* | Casuarinaceae | Tree | Kokubugata and Yokota, 2012 |
| *C. filiformis* | Japan | *Chamaesyce atoto* | Euphorbiaceae | Herb | Kokubugata and Yokota, 2012 |
| *C. filiformis* | Japan | *Chrysanthemum crassum* | Asteraceae | Herb | Kokubugata and Yokota, 2012 |
| *C. filiformis* | Japan | *Cinnamomum doederleinii* | Lauraceae | Tree | Kokubugata and Yokota, 2012 |
| *C. filiformis* | Japan | *Clerodendrum inerme* | Verbenaceae | Shrub | Kokubugata and Yokota, 2012 |
| *C. filiformis* | Japan | *Dicranopteris linearis* | Gleicheniaceae | Fern | Kokubugata and Yokota, 2012 |
| *C. filiformis* | Japan | *Dodonaea viscosa* | Sapindaceae | Shrub | Kokubugata and Yokota, 2012 |
| *C. filiformis* | Japan | *Gymnosporia diversifolia* | Celastraceae | Shrub or tree | Kokubugata and Yokota, 2012 |
| *C. filiformis* | Japan | *Heteosmilax japonica* | Smilacaceae | Vine | Kokubugata and Yokota, 2012 |
| *C. filiformis* | Japan | *Ipomoea pes-caprae* | Convolvulaceae | Vine | Kokubugata and Yokota, 2012 |
| *C. filiformis* | Japan | *Ischaemum aristatum* | Poaceae | Herb | Kokubugata and Yokota, 2012 |
| *C. filiformis* | Japan | *Lysimachia mauritiana* | Primulaceae | Herb | Kokubugata and Yokota, 2012 |
| *C. filiformis* | Japan | *Melanthera biflora* | Asteraceae | Herb | Kokubugata and Yokota, 2012 |
| *C. filiformis* | Japan | *Pandanus odoratssimus* | Pandanaceae | Tree | Kokubugata and Yokota, 2012 |
| *C. filiformis* | Japan | *Peucedanum japonicum var. japonicum* | Apiaceae | Herb | Kokubugata and Yokota, 2012 |
| *C. filiformis* | Japan | *Scaevola taccada* | Goodeniaceae | Shrub | Kokubugata and Yokota, 2012 |
| *C. filiformis* | Japan | *Spinifex littoreus* | Poaceae | Herb | Kokubugata and Yokota, 2012 |
| *C. filiformis* | Japan | *Thrarea involuta* | Poaceae | Herb | Kokubugata and Yokota, 2012 |
| *C. filiformis* | Japan | *Vigna marina* | Fabaceae | Vine | Kokubugata and Yokota, 2012 |
| *C. filiformis* | Japan | *Vitex rotundifolia* | Verbenaceae | Shrub | Kokubugata and Yokota, 2012 |
| *C. filiformis* | Japan | *Zanthoxylum beecheyanum var. alatum* | Rutaceae | Shrub | Kokubugata and Yokota, 2012 |
| *C. filiformis* | Japan | *Zoysia pacifica* | Poaceae | Herb | Kokubugata and Yokota, 2012 |
| *C. filiformis* | Mediterranean areas | *Bidens pilosa* | Asteraceae | Herb | WIKTROP |
| *C. filiformis* | Pakistan | *Bougainvillea spectabilis* | Nyctaginaceae | Shrub | Mukhtar et al., 2010 |
| *C. filiformis* | Pakistan | *Butea monosperma* | Fabaceae | Tree | Mukhtar et al., 2010 |
| *C. filiformis* | Pakistan | *Citrus aurantifolia* | Rutaceae | Tree | Mukhtar et al., 2010 |
| *C. filiformis* | Pakistan | *Morus alba* | Moraceae | Tree | Mukhtar et al., 2010 |
| *C. filiformis* | Pakistan | *Nerium oleander* | Apocynaceae | Shrub | Mukhtar et al., 2010 |
| *C. filiformis* | Pakistan | *Thevetia peruviana* | Apocynaceae | Tree | Mukhtar et al., 2010 |
| *C. filiformis* | Pakistan | *Ziziphus manritiana* | Rhamnaceae | Tree | Mukhtar et al., 2010 |
| *C. filiformis* | Tanzania | *Acacia sieberiana* | Fabaceae | Tree | Buriyo et al., 2015 |
| *C. filiformis* | Tanzania | *Albizia gummifera* | Fabaceae | Tree | Buriyo et al., 2015 |
| *C. filiformis* | Tanzania | *Albizia lebbeck* | Fabaceae | Tree | Buriyo et al., 2015 |
| *C. filiformis* | Tanzania | *Albizia petersiana* | Fabaceae | Tree | Buriyo et al., 2015 |
| *C. filiformis* | Tanzania | *Anacardium occidentale* | Anacardiaceae | Tree | Buriyo et al., 2015 |
| *C. filiformis* | Tanzania | *Artocarpus heterophylla* | Moraceae | Tree | Buriyo et al., 2015 |
| *C. filiformis* | Tanzania | *Azadirachta indica* | Meliaceae | Tree | Buriyo et al., 2015 |
| *C. filiformis* | Tanzania | *Brachystegia spiciformis* | Fabaceae | Tree | Buriyo et al., 2015 |
| *C. filiformis* | Tanzania | *Canthium zanzibaricum* | Rubiaceae | Shrub | Buriyo et al., 2015 |
| *C. filiformis* | Tanzania | *Citrus sinensis* | Rutaceae | Tree | Buriyo et al., 2015 |
| *C. filiformis* | Tanzania | *Dicrostachyus cinerea* | Fabaceae | Tree | Buriyo et al., 2015 |
| *C. filiformis* | Tanzania | *Dombeya rotundjfolia* | Malvaceae | Tree | Buriyo et al., 2015 |
| *C. filiformis* | Tanzania | *Ficus sur* | Moraceae | Tree | Buriyo et al., 2015 |
| *C. filiformis* | Tanzania | *Flueggia virosa#* | ？ | ？ | Buriyo et al., 2015 |
| *C. filiformis* | Tanzania | *Grewia microcarpa* | Malvaceae | Herb | Buriyo et al., 2015 |
| *C. filiformis* | Tanzania | *Hymenocardia ulmoides* | Phyllanthaceae | Tree | Buriyo et al., 2015 |
| *C. filiformis* | Tanzania | *Hyparrhenia rufa* | Poaceae | Herb | Buriyo et al., 2015 |
| *C. filiformis* | Tanzania | *Lannea schimperi* | Anacardiaceae | Shrub or tree | Buriyo et al., 2015 |
| *C. filiformis* | Tanzania | *Lannea stuhjmanii* | Anacardiaceae | Shrub or tree | Buriyo et al., 2015 |
| *C. filiformis* | Tanzania | *Lantana camara* | Verbenaceae | Shrub | Buriyo et al., 2015 |
| *C. filiformis* | Tanzania | *Lawsonia inermis* | Lythraceae | Tree | Buriyo et al., 2015 |
| *C. filiformis* | Tanzania | *Maclura africana* | Moraceae | Shrub or tree | Buriyo et al., 2015 |
| *C. filiformis* | Tanzania | *Mangifera indica* | Anacardiaceae | Tree | Buriyo et al., 2015 |
| *C. filiformis* | Tanzania | *Ochna mossambicensis* | Ochnaceae | Tree | Buriyo et al., 2015 |
| *C. filiformis* | Tanzania | *Ozoroa mucronata* | Anacardiaceae | Fern | Buriyo et al., 2015 |
| *C. filiformis* | Tanzania | *Pluchea dioscorides* | Asteraceae | Shrub | Buriyo et al., 2015 |
| *C. filiformis* | Tanzania | *Pteliopsis myritifolia* | Combretaceae | Tree | Buriyo et al., 2015 |
| *C. filiformis* | Tanzania | *Sesbania sesban* | Fabaceae | Tree | Buriyo et al., 2015 |
| *C. filiformis* | Tanzania | *Spirostachys africana* | Euphorbiaceae | Tree | Buriyo et al., 2015 |
| *C. filiformis* | Tanzania | *Syzygium cumini* | Myrtaceae | Tree | Buriyo et al., 2015 |
| *C. filiformis* | Tanzania | *Tamarin dusindica* | Fabaceae | Tree | Buriyo et al., 2015 |
| *C. filiformis* | Tanzania | *Trema orientalis* | Ulmaceae | Tree | Buriyo et al., 2015 |
| *C. filiformis* | Tanzania | *Vernonia amygdalina* | Asteraceae | Shrub | Buriyo et al., 2015 |
| *C. filiformis* | Tanzania | *Vitex doniana* | Lamiaceae | Tree | Buriyo et al., 2015 |
| *C. filiformis* | United States | *Schinus terebinthifolius* | Anacardiaceae | Shrub | Burch, 1992; Musselman, 1996; Manrique et al., 2009 |
| *C. filiformis* | Not available | *Eugenia aromatica (cloves)* | Myrtaceae | Tree | Nelson, 2008 |
| *C. filiformis* | Not available | *Myristica fragrans (nutmeg)* | Myristicaceae | Tree | Nelson, 2009 |
| *C. filiformis* | Not available | *Persea americana (avocado)* | Lauraceae | Tree | Nelson, 2010 |
| *C. filiformis* | Not available | *Hyparrhenia* spp.* | Poaceae | Herb | Buriyo et al., 2015 |
| ***C. flava*** | Australia | *Pileanthus filifolius* | Myrtaceae | Shrub | Perry et al., 2009 |
| ***C. glabella*** | Australia | *Banksia marginata* | Proteaceae | Shrub or tree | Ziegler, 1995 |
| *C. glabella* | Australia | *Casuarina equisetifolia* | Casuarinaceae | Tree | Ziegler, 1995 |
| *C. filiformis* | India | *Trema orientalis** | Ulmaceae | Tree | Debabrata 2018 |
| *C. glabella* | Japan | *Aristida takeoi* | Poaceae | Herb | Kokubugata and Yokota, 2012 |
| *C. glabella* | Japan | *Rhynchospora rubra* | Cyperaceae | Herb | Kokubugata and Yokota, 2012 |
| *C. filiformis* | China | *Lantana camara** | Verbenaceae | Shrub | Li et al., 1992 |
| *C. melantha* | Australia | *Acacia melanoxylon* | Fabaceae | Tree | Ziegler, 1995 |
| *C. filiformis* | India | *Lantana camara** | Verbenaceae | Shrub | Debabrata 2018 |
| ***C. pomiformis*** | Australia | *Lamarchea hakeifolia* | Myrtaceae | Shrub | Hardman and Moro, 2006 |
| *C. pomiformis* | Australia | *Melaleuca huegelii subsp. pristicensis* | Myrtaceae | Shrub | O'Neill et al., 2021 |
| ***C. pubescens*** | Australia | *Acacia myrtifolia* | Fabaceae | Shrub | Facelli et al., 2020 |
| *C. pubescens* | Australia | *Acacia paradoxa* | Fabaceae | Shrub | Cirocco et al., 2017, 2021 |
| *C. pubescens* | Australia | *Cytisus scoparius* | Fabaceae | Shrub | Facelli et al., 2020 |
| *C. pubescens* | Australia | *Leptomeria scoparium* | Myrtaceae | Shrub | Close et al., 2006 |
| *C. pubescens* | Australia | *Leptospermum myrsinoides* | Myrtaceae | Shrub | Ziegler, 1995; Facelli et al., 2020 |
| *C. pubescens* | Australia | *Ulex europaeus* | Fabaceae | Shrub | Cirocco et al., 2017; Facelli et al., 2020 |
| *C. pubescens* | Denmark | *Pavonia praemorsa* | Malvaceae | Shrub | Heide-Jørgensen, 1991 |
| *C. pubescens* | Denmark | *Hibiscus rosasinensis* | Malvaceae | Shrub or tree | Heide-Jørgensen, 1991 |

* Genus level or repeated species in different countries.

# Species name that not found from the Plant List.

Note: we used the species name from the references, some of which may be not right. The family names were checked according to the Plants of the World Online (POWO).

REFERENCES

Abubacker, M. N., Prince, M., and Hariharan, Y. (2005). Histochemical and

biochemical studies of parasite-host interaction of Cassytha filiformis Linn. and

Zizyphus jujuba Lamk. Curr. Sci. 89, 2156–2159.

Burch, J. N. (1992). Cassytha filiformis and limits to growth and reproduction of

Schinus terebinthifolius in southern Florida. Fla. Sci. 55, 28–34.

Hardman, B., and Moro, D. (2006). Importance of diurnal refugia to a hare-wallaby

reintroduction in Western Australia. Wildl. Res. 33, 355–359. doi: 10.1071/

WR05088

Ho, J. C., Chen, C. M., and Row, L. C. (2004). Neolignans from the parasitic plants.

Part 2. Cassytha filiformis. J. Chin. Chem. Soc. 51, 221–223. doi: 10.1002/jccs.

200400034

Mukhtar, I., Khokhar, I., and Mushtaq, S. (2010). First report on Cassytha filiformis

L. (Lauraceae), a parasitic weed from Lahore, Pakistan. Pak. J. Weed Sci. Res. 16,

451–457.

O’Neill, S., Short, J., and Calver, M. (2021). The distribution, habitat preference

and population dynamics of the pale field-rat (Rattus tunneyi) at Edel Land,

Shark Bay, Western Australia: the role of refuges and refugia in population

persistence.Wildl. Res. 48, 444–457. doi: 10.1071/WR20005

Perry, G. L. W., Enright, N. J., Miller, B. P., and Lamont, B. B. (2009). Nearestneighbour

interactions in species-rich shrublands: the roles of abundance,

spatial patterns and resources. Oikos 118, 161–174. doi: 10.1111/j.1600-0706.

2008.16947.x

Ren, H., Jian, S. G., Zhang, Q. M., Wang, F. G., Shen, T., and Wang, J. (2017). Plants

and vegetation on south China sea islands. Ecol. Environ. Sci. 26, 1639–1648.
